# Systems Pharmacology Dissection of Cholesterol Regulation Reveals Determinants of Large Pharmacodynamic Variability between Cell Lines

**DOI:** 10.1016/j.cels.2017.11.002

**Published:** 2017-12-27

**Authors:** Peter Blattmann, David Henriques, Michael Zimmermann, Fabian Frommelt, Uwe Sauer, Julio Saez-Rodriguez, Ruedi Aebersold

**Affiliations:** 1 Department of Biology, Institute of Molecular Systems Biology, ETH Zurich, Auguste-Piccard-Hof 1, 8093 Zurich, Switzerland; 2 IIM-CSIC Spanish Council for Scientific Research, (Bio)Process Engineering Group, C/Eduardo Cabello 6, 36208 Vigo, Spain; 3 RWTH-Aachen University, Faculty of Medicine, Joint Research Centre for Computational Biomedicine (JRC-COMBINE), MTZ Pauwelstrasse 19, D-52074 Aachen, Germany; 4 European Molecular Biology Laboratory, European Bioinformatics Institute, Wellcome Trust Genome Campus, Hinxton, Cambridge CB10 1SD, UK; 5 Faculty of Science, University of Zurich, Zurich, Switzerland

**Keywords:** proteomics, metabolomics, mass spectrometry, logic modeling, statins, T0901317, GW3965, LXR, SREBP, SWATH

## Abstract

In individuals, heterogeneous drug-response phenotypes result from a complex interplay of dose, drug specificity, genetic background, and environmental factors, thus challenging our understanding of the underlying processes and optimal use of drugs in the clinical setting. Here, we use mass-spectrometry-based quantification of molecular response phenotypes and logic modeling to explain drug-response differences in a panel of cell lines. We apply this approach to cellular cholesterol regulation, a biological process with high clinical relevance. From the quantified molecular phenotypes elicited by various targeted pharmacologic or genetic treatments, we generated cell-line-specific models that quantified the processes beneath the idiotypic intracellular drug responses. The models revealed that, in addition to drug uptake and metabolism, further cellular processes displayed significant pharmacodynamic response variability between the cell lines, resulting in cell-line-specific drug-response phenotypes. This study demonstrates the importance of integrating different types of quantitative systems-level molecular measurements with modeling to understand the effect of pharmacological perturbations on complex biological processes.

## Highlights

• Mass spectrometry-based quantification of multi-level cellular response to drugs
• Generation of a large multi-omics dataset from different perturbed cell lines
• Capturing the cell-type-specific responses to drugs with mathematical models
• Identification of pharmacodynamic variability as major determinant of drug response

## Graphical Abstract

**Figure:**
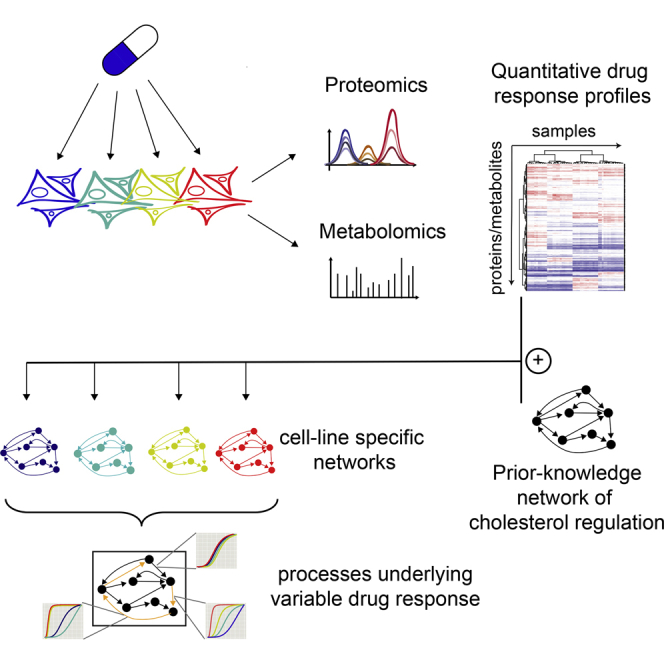

## Teaser

Blattmann et al. combine proteomic and metabolomic profiling with mechanistic modeling to characterize the complex processes underlying the heterogeneous drug response in cellular cholesterol regulation across a panel of different human cell lines.

## Introduction

The molecular response of cells and tissues to drugs is complex, and the same drug can elicit varying response phenotypes (response, toxicity, or lack of response) in individual patients (Berg et al., 2010, Meyer et al., 2013). Prediction of personalized drug response is therefore a key issue of personalized/precision medicine and is expected to require mathematical models to optimally predict outcome (Berg et al., 2010, Bordbar et al., 2015, van Hasselt and van der Graaf, 2015, Xie et al., 2016). However, even in pre-clinical model systems, such as individual cell lines, it remains challenging to predict the variability in drug response (Iorio et al., 2016). Despite having a good qualitative understanding about a wide range of cellular processes, we do not fully understand which processes are responsible, how much they contribute to the variable drug response, and how they are quantitatively modulated by a specific treatment regimen. Hence, we require new advances in our strategies of collecting informative molecular data and extracting the relevant information.

A large, community-based study showed that mathematical models that rely on several data types and used prior biological knowledge performed best in predicting drug response (Costello et al., 2014). To date, the input data to build personalized or cell-line-specific models have consisted, for the most part, of genomic mutations and baseline transcript or metabolite abundance (Bordbar et al., 2015, Iorio et al., 2016). The availability of only baseline data, however, limits the ability to characterize the variable molecular drug-response mechanisms, which would require quantifying the cellular biomolecules before and after drug treatment (Abelin et al., 2016, Lamb et al., 2006). Furthermore, quantifying proteins and metabolites, as opposed to transcripts, might be superior in detecting the subtle differences in the variable drug response, as these molecules are closer to the observed phenotype (Liu et al., 2016). Indeed, protein abundance data add additional information to samples analyzed previously by sequencing and measuring transcript abundance (Mertins et al., 2016). Recently, mass spectrometry (MS)-based methods have become available to measure metabolite and protein abundances accurately and reliably on a large scale (Fuhrer et al., 2011, Gillet et al., 2016).

Here, we explore how proteomic and metabolic data acquired before and after various drug treatments can be used to train mathematical models that describe mechanistically the heterogeneous drug response across a panel of cell lines. We selected cholesterol regulation as a prototypical example of a druggable complex biological process for the following three reasons: first, cholesterol homeostasis is clinically highly relevant as its dysregulation is a major risk factor for cardiovascular disease, non-alcoholic fatty liver disease, and cancer (Moon et al., 2012, Shao and Espenshade, 2012). Second, extensive prior mechanistic knowledge is available from decades of research (Brown and Goldstein, 2009). Third, a number of clinically used and experimental drugs are available to perturb the system. The process of cholesterol regulation is essentially a biochemical feedback control system. When the cholesterol concentration in the membrane of the endoplasmic reticulum (ER) drops below a critical level (Radhakrishnan et al., 2008), sterol regulatory element-binding proteins (SREBPs: SREBP1a/1c/2) are activated and trigger an increased expression of SREBP target proteins, which results in a rapid normalization of the cholesterol levels in the ER (Brown and Goldstein, 2009, Horton et al., 2003). Statins reduce the risk for cardiovascular disease by inhibiting cholesterol synthesis. This triggers a whole cascade of cellular processes starting with the activation of SREBP. Despite a large degree of inter-individual heterogeneity in the blood lipid-lowering effects of statins (Chasman et al., 2012, Mangravite et al., 2006), the currently known and replicated genetic factors only explain a small part of the variability (Leusink et al., 2016, Theusch et al., 2016). Liver X receptor (LXR) is another important transcription factor in cellular cholesterol regulation and controls the expression of proteins involved in reverse cholesterol transport from peripheral cells to the liver (Calkin and Tontonoz, 2012). Both SREBP and LXR have been studied extensively, but no systematic assessment of the effects of SREBP and LXR perturbation on protein and metabolite abundance has been performed. Hence, it is unclear how a variable genetic background affects the drug response mediated by these two transcription factors.

To study the heterogeneity in the cellular drug-response phenotypes, cholesterol regulation was perturbed in up to 23 different conditions in each of the four human cell lines HEK293, HeLa Kyoto, Huh7, and HepG2. The perturbations included treatment with atorvastatin, two LXR agonists (T0901317 and GW3965), 25-hydroxycholesterol, lipoprotein-deficient serum (LPDS) and a range of small interfering RNAs (siRNAs) directed at components of the cholesterol homeostasis system. Following these perturbations, the proteomic response was quantified for all 23 conditions, the metabolomic response for the 12 drug-treated conditions, and the phosphoproteomic response for two drug-treated conditions. Collectively, the measurement of these 491 different samples resulted in the quantification of up to 6,000 different metabolites, proteins, or phosphopeptides. The data were used to train cell-line-specific models of cellular cholesterol regulation that describe in detail how the cells responded differently to the same perturbation. The models revealed that although the intracellular drug amount was an important determinant for drug response within the same cell line, the heterogeneity in drug response between different cells strongly depended on several pharmacodynamic differences.

## Results

### MS-Based Acquisition of Proteomic and Metabolic Drug-Response Profiles

**Figure 1:**
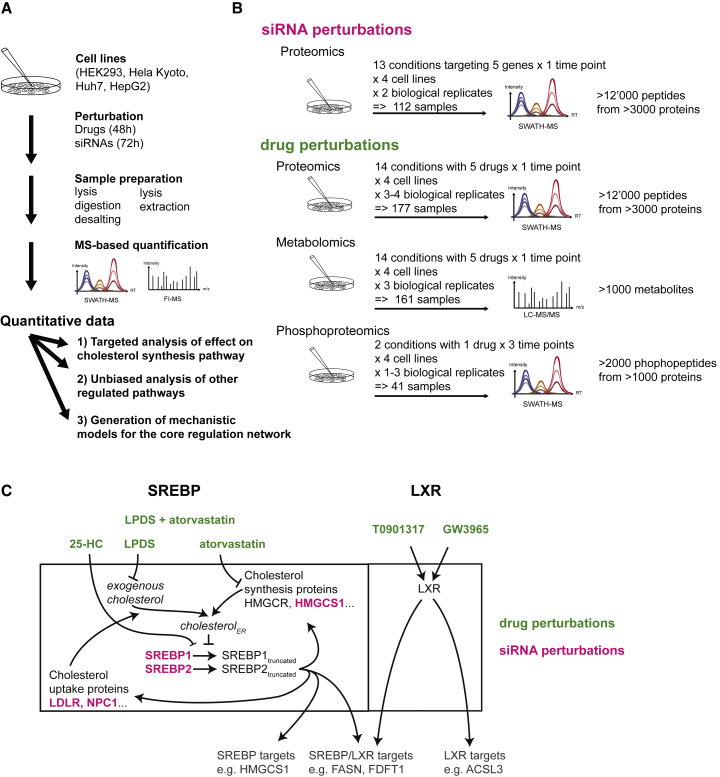
Experimental Workflow and Core Regulation Network of Cholesterol Homeostasis (A) Experimental workflow. (B) Study design. (C) Core regulation model of cellular cholesterol regulation depicting the positive (arrows) or negative (⊣) functional interactions, and representing the perturbations in color (drug, green; siRNA, magenta). 25-HC, 25-hydroxy-cholesterol; LPDS, lipoprotein-deficient plasma serum.

The cellular heterogeneity in drug response was characterized by quantitatively measuring protein and metabolite profiles after perturbing cellular cholesterol homeostasis in a panel of four genetically different human cell lines (Huh7, HepG2, HEK293, and HeLa Kyoto), frequently used to analyze cholesterol regulation (Figures 1A and 1B) (Blattmann et al., 2013, Horton et al., 2002, Medina et al., 2012, Xu et al., 2015). In particular, the human liver-derived Huh7 and HepG2 cells represent well-established model cell lines for testing drug response and metabolism (Ahlin et al., 2009). Cholesterol homeostasis was perturbed using drugs and siRNAs that affected the two main transcription factors of cellular cholesterol regulation (SREBP and LXR) in different ways (Figure 1C). The 23 perturbations for each cell line were performed in biological triplicates or duplicates using different drug concentrations and two independent siRNAs per gene (Figures 1B and 1C). To reach a quasi steady-state, cells were treated for 48 hr with drugs, or for 72 hr with siRNAs, and protein and metabolite abundances were measured using MS. For the genetic perturbations solely proteins were measured. Enriched phosphopeptides were measured for one perturbation (LPDS + 1 μM atorvastatin) (Figure 1B). This resulted in 330 samples for which peptides were quantified, and 161 samples for which the metabolites were extracted and measured.

**Figure 2:**
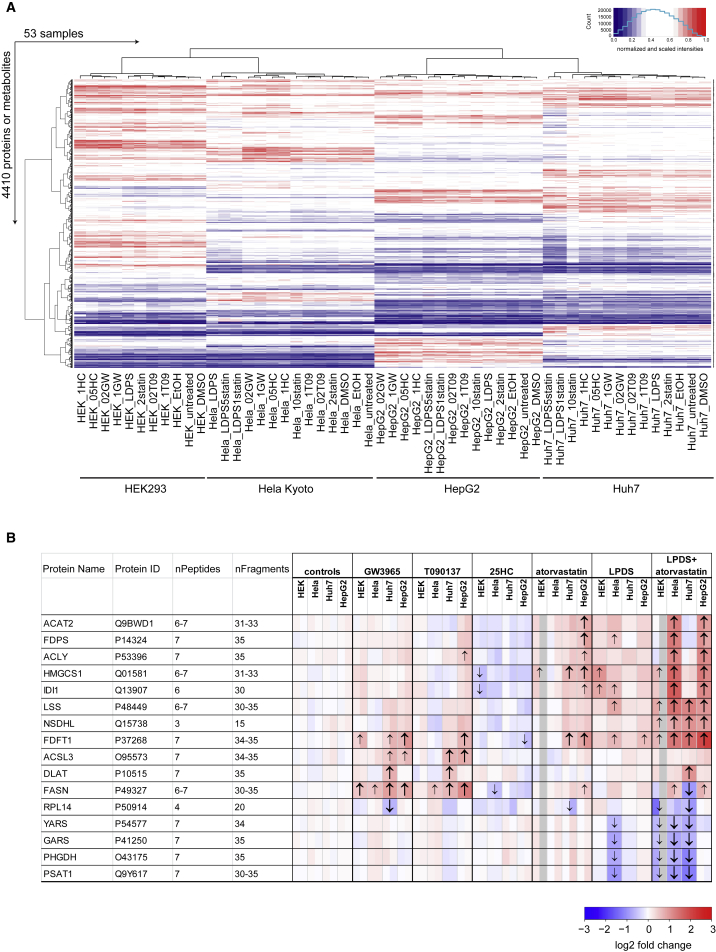
Quantitative Results of Differentially Abundant Metabolites and Proteins (A) Abundances of proteins and metabolites after drug perturbations (sample labels are explained in the STAR Methods; see also Figure S1). (B) Heatmap of the 16 proteins differentially expressed in >6 conditions. The arrows indicate the directionality of a significant change in expression (n = 3; |log2FC| > 0.5 and FDR < 0.001). Small arrows indicate a significant change in only one drug concentration, large bold arrows in both concentrations. For further details see the STAR Methods. For Metabolites see Figure S2.

The proteomic response to treatments was quantified using a data-independent acquisition approach (SWATH-MS), a massively parallel MS-based peptide quantification technique with the capacity to accurately quantify and reproducibly detect tens of thousands of peptides in a single injection (Gillet et al., 2012). The metabolic response was measured using a flow-injection MS platform, which allowed untargeted quantification of more than 1,000 metabolites (Fuhrer et al., 2011). The reproducibility of the measurements for both metabolites and proteins across the biological replicates was high (R^2^ = 0.73–0.98), even for those acquired on different instruments several months apart. The variation of the signal across treatments was at least twice as high as the measurement error between biological replicates (see the STAR Methods). These techniques allowed the quantification of >12,000 peptides from >3,000 different proteins, >1,000 metabolites, and >2,000 phosphopeptides with high consistency across the samples and resulted in a very large data resource (Tables S1, S2, and S3). For example, the abundance of 3,364 proteins and 1,046 metabolites measured across 53 drug-treated samples in biological triplicates described in detail the molecular drug-response phenotypes (Tables S1 and S2; Figure 2A).

Hierarchical clustering of the molecular profiles showed that the samples from the same cell line clustered together and within these clusters the samples treated with different concentrations of the same drug typically clustered together (Figures 2A and S1). These clusters indicated that each cell line had a unique initial biomolecular profile that was modulated in a cell-specific manner in response to the treatments. In total, 42% (435) of the metabolite abundances, 21% (694) of the protein abundances, and 25% (525) of the phosphopeptide abundances were significantly different in one or more drug-treated condition compared to the control samples of the same cell line (a log2 transformed fold change |log2FC| > 0.5 and a false discovery rate [FDR] < 0.01) (Tables S1, S2, and S3). However, only the abundance of few molecules differed significantly across many conditions or cell lines: 16 proteins were differently expressed in at least 6 different drug-treated samples (Figure 2B), 18 metabolites differed in abundance in at least 10 drug-treated conditions (Figure S2A), and the abundance of 31 metabolites differed in at least 4 LXR agonist-treated conditions (Figure S2B). The abundance of phosphopeptides from 54 proteins was affected in at least 2 cell lines (Figure S3C). Overall, the MS analyses generated extensive quantitative protein and metabolite profiles that reflected the complex drug-response phenotypes among our panel of cell lines (Figures 2A and S1; Tables S1, S2, and S3). First, the observed effects were related to the existing knowledge of the literature-based core regulation model (Figure 1C) and, in a second step, cell-line-specific models were generated to explain the drug response and identify the underlying variable mechanisms.

### The Core Cellular Cholesterol Regulation Model Is Functional in All Cell Lines

**Figure 3:**
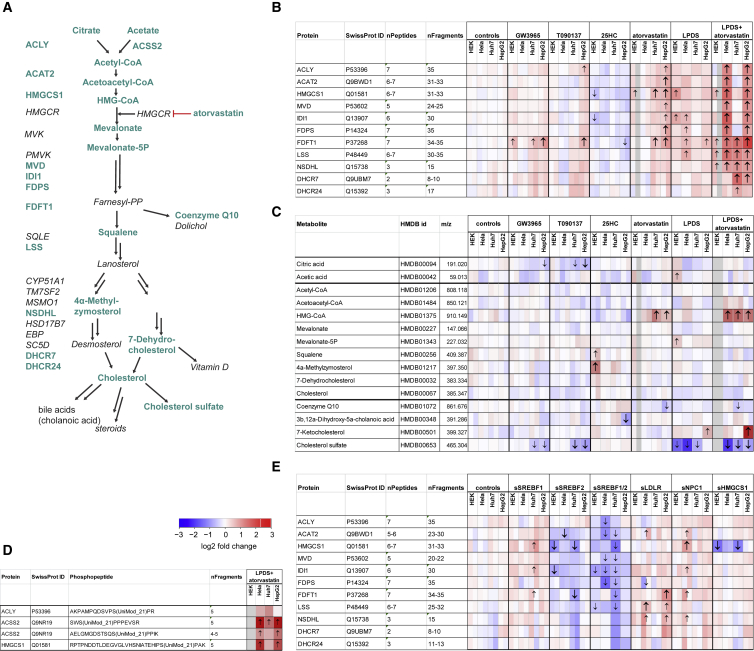
Quantitative Data for the Cholesterol Synthesis Pathway (A) Cholesterol synthesis pathway with quantified proteins and metabolites labeled in color. (B) Heatmap showing the difference in expression for the cholesterol synthesis enzymes. (C) Heatmap showing the difference in abundance of the metabolites from the cholesterol synthesis pathway. (D) Heatmap showing the difference in abundance of phosphopeptides after LPDS + 1 μM atorvastatin treatment. One of the probable localization of the HMGCS1 phosphorylation site is shown (see also Table S3 and Figure S3). (E) Heatmap showing difference in expression of the cholesterol synthesis enzymes after treatment with siRNAs. (B–E) Arrows indicate the direction of the statistically significant change in expression: (B and E) n = 3; |log2FC| > 0.5 and FDR < 0.001; (C and D) n = 3; |log2FC| > 0.5 and FDR < 0.01. For further details, see the STAR Methods.

**Figure 4:**
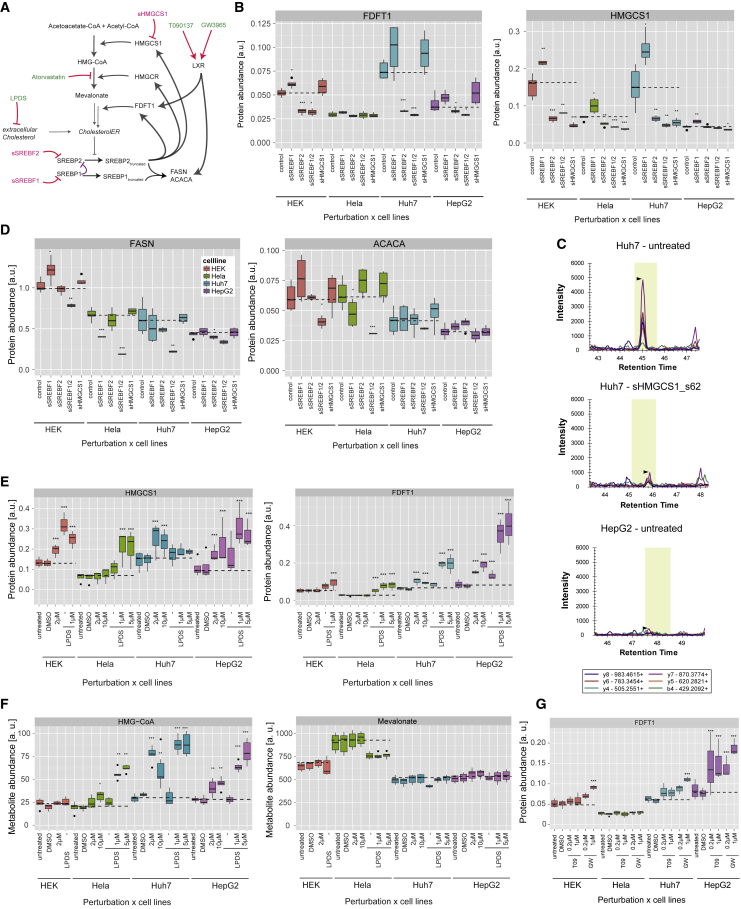
Core Regulatory Mechanisms (A) Core regulatory mechanisms relevant for this figure. Depicted in violet is a hypothetical inhibitory interaction between SREBP1 and SREBP2. (B and D) Protein abundance upon knock down of key regulators. Significance of differential expression was tested by combining the measurements for the different siRNAs and using an unpaired t test. n = 2–6; ^∗^p < 0.1, ^∗∗^p < 0.05, ^∗∗∗^p < 0.01. (C) Signal extracted for different fragments of the NNLSYDC[+57]IGR peptide from HMGCS1 with the software Skyline (MacLean et al., 2010). The yellow area shows the predicted retention time, and the black arrowheads indicate the peak. (E) Abundance of HMGCS1 and FDFT1 upon drug treatment. (F) Metabolite levels for HMG-CoA and mevalonate upon drug treatment. (G) Protein levels of FDFT1 upon activation of LXR. (E–G) Significant differential expression was reached with |log2FC| > 0.5 and adjusted ^∗^p < 0.1, ^∗∗^p < 0.01, ^∗∗∗^p < 0.001 compared with control samples. For other regulated pathways, see Figures S4 and S5.

Based on the current knowledge about cholesterol regulation (Brown and Goldstein, 2009), a literature-based core regulation model was generated that explains how cholesterol homeostasis is maintained in cells (Figure 1C). Cholesterol synthesis is regulated mainly by the transcription factor SREBP2 but also by SREBP1 (Amemiya-Kudo et al., 2002, Horton et al., 2003). In our cell lines, these enzymes were regulated predominantly by SREBP2, as only knock down of *SREBF2*, but not *SREBF1*, decreased their expression (Figures 3E and 4B). Enzymes responsible for lipid synthesis (FASN and ACACA) and known to be regulated by SREBP1 were reduced upon combined *SREBF2* and *SREBF1* knockdown (Figure 4D).

According to the core regulation model, perturbations that reduce either cholesterol uptake (LPDS, siLDLR, and siNPC1) or cholesterol synthesis (atorvastatin and siHMGCS1) should lead to an activation of SREBP2 and an increased expression of cholesterol synthesis enzymes (Figure 1C). Conversely, treatment with 25-hydroxycholesterol and siRNA-mediated knock down of SREBP2 (siSREBF2) would reduce the activity of SREBP2 and the abundance of cholesterol synthesis enzymes. The change in abundance of ten cholesterol synthesis enzymes and the preceding enzymes ACLY and ACSS2 (Figure 3A) were, except for HMGCS1 knockdown, as predicted by the core regulation model (Figures 3B and 3E). All enzymes passed our stringent criteria for differential expression (|log2FC| > 0.5 and FDR <0.001 against all negative controls) in at least one perturbation and in 4.3 out of the 9 perturbations on average. Notably, inhibition of either cholesterol uptake (LPDS) or synthesis (atorvastatin) resulted in weaker effects than the simultaneous inhibition of both processes (LPDS + atorvastatin) (Figure 3B), suggesting that all cells were capable of both cholesterol synthesis and uptake and that these processes could partially compensate for each other. The only perturbation that showed no significant effect on this pathway was the siRNA-mediated knock down of HMGCS1 (Figure 3E) which, despite a 60% reduction in HMGCS1 protein levels in Huh7 (FC = 0.39, FDR < 7.3 × 10^−13^) and HEK293 cells (FC = 0.39, FDR <5.9 × 10^−17^) (Table S1; Figure 4C), did not elicit a significant response (Figure 3E). That even a considerable loss in HMGCS1 activity did not activate SREBP2 indicated that HMGCS1 is not a rate-limiting enzyme in this pathway, a finding in agreement with the general view that HMGCR catalyzes the committing and first rate-limiting step (Brown and Goldstein, 2009). In HeLa and HepG2 cells no significant reduction in HMGCS1 abundance could be measured because the basal expression in untreated cells was already close to the limit of detection (Figure 4C).

The effect of sterol depletion on the cholesterol synthesis pathway was compared for the different molecule types (proteins, metabolites, and phosphopeptides) (Figures 3B–3D). Although many enzyme levels and several phosphopeptide levels were regulated (Figures 3B and 3D), 3-hydroxy-3-methylglutaryl coenzyme-A (HMG-CoA) was the only one, out of nine quantified metabolites of this pathway, which consistently changed its abundance (e.g., LPDS + statin in Huh7 cells, mean FC = 2.81, adjusted p < 9.5 × 10^−5^) (Table S2; Figure 3C). As HMG-CoA is the substrate of HMGCR, the accumulation of HMG-CoA indicated that HMGCR was efficiently inhibited by the statin treatment (Figure 3A). Despite the reduced biosynthetic capacity, the other metabolites maintained their steady-state levels (Figures 3C and 4F). As the relevant cholesterol pool in the ER regulated by these perturbations accounts for <1% of the total cellular cholesterol (Lange et al., 1999), no regulation of the total cellular cholesterol was observed (Figure 3C). However, the levels of the downstream metabolite cholesterol sulfate were reduced (e.g., cholesterol sulfate: LPDS + statin in Huh7 cells, mean FC = 0.54, adjusted p < 5.6 × 10^−6^), suggesting that cholesterol sulfate might serve as a readout for cholesterol depletion (Table S2; Figure 3C).

In summary, the complementary protein and metabolite data demonstrate that the treatments successfully perturbed cholesterol regulation in agreement with previous canonical knowledge (Figure 1C), and that cholesterol uptake, cholesterol synthesis, the feedback by SREBP2, and inhibition by statins were conserved and functional in all cell lines tested.

### Complementation for SREBP1 by SREBP2

In addition we also observed responses that have so far not been well characterized. For example, knockdown of *SREBF1* resulted in a significantly increased abundance of the SREBP2 target proteins FDFT1, HMGCS1, and IDI1 in Huh7 cells (Table S1; Figures 3E and 4B), and showed a trend for increased expression of other cholesterol synthesis enzymes in HepG2, HEK293, and HeLa cells (Table S1; Figures 3E and 4B). That depletion of *SREBF1* is compensated by an increased abundance of SREBP2 has, to our knowledge, only been reported once in the liver of SREBP1 knockout mice (Shimano et al., 1997). Our results support these previous findings but add that (1) this mechanism does not require an organismal context, (2) that such a mechanism seems to be conserved across various cell lines, and (3) that this mechanism can be activated by a transient depletion of SREBP1 by siRNAs.

### Effect of LXR on the Cholesterol Synthesis Pathway

There have been conflicting reports about the effect of LXR activation on the cholesterol synthesis pathway. Schultz et al. (2000) showed that treatment of mice with the LXR agonist T0901317 reduced hepatic mRNA levels of both HMGCS1 and FDFT1, and Wang et al. (2008) reported increased FDFT1 protein levels upon siRNA-mediated depletion of LXRα in HepG2 cells. In contrast, a profiling study of the transcriptome of THP-1 cells found that FDFT1 mRNA expression was increased upon T090137 treatment (Pehkonen et al., 2012). FDFT1 (squalene synthase) is a crucial enzyme as it catalyzes the committed step after the last branchpoint in the cholesterol synthesis pathway (Figure 3A), and pharmacologically inhibiting FDFT1 can reduce blood LDL levels (Stein et al., 2011). Therefore, it is important to understand the directionality of the effect of LXR activation on FDFT1 levels. In our study, the quantification of FDFT1 with more than 30 fragment ions from 7 proteotypic peptides showed a significant increase in FDFT1 protein expression in HepG2 cells after treatment with 0.2 and 1 μM T090137 and GW3965, and in Huh7 and HEK293 cells after treatment with 1 μM GW3965 (Table S1; Figure 4G). Concomitantly, the expression levels of several other enzymes in the cholesterol synthesis pathway were slightly increased upon LXR stimulation in HepG2 and Huh7 cells (Figure 3B). Collectively, our data thus suggest a positive directionality between LXR activation, cholesterol synthesis, and FDFT1 expression.

### Differential Regulation of the First Part of the Mevalonate Pathway in Huh7 Cells

Surprisingly, cholesterol depletion with LPDS and atorvastatin in Huh7 cells did not significantly increase the levels of the enzymes upstream of farnesyl-PP (ACLY, ACAT2, HMGCS1, MVD, IDI1, and FPDS) (Figure 3B). In contrast, the same treatment in HepG2 and HeLa cells resulted in strongly increased expression of these enzymes (Figure 3B) as observed previously by selected reaction monitoring (Kusebauch et al., 2016).

**Figure 5:**
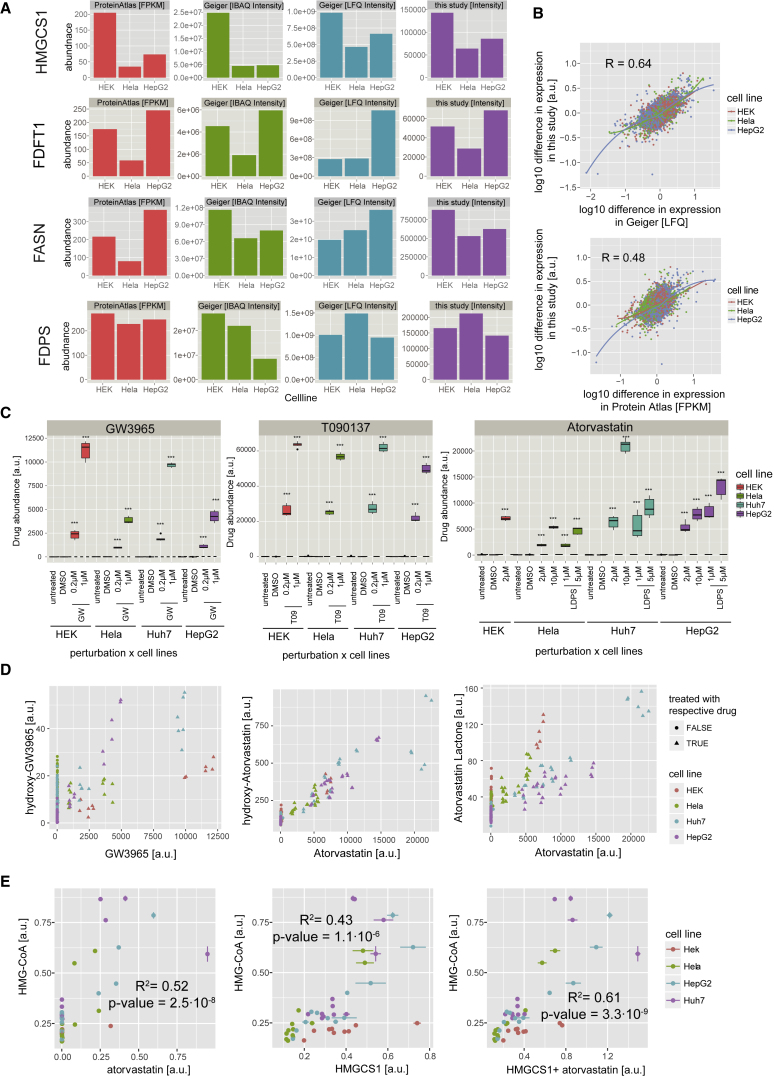
Heterogeneity in Basal Protein Expression, Drug Disposition, and Drug Response (A) Comparison of basal expression in three cell lines (Huh7 cells were not quantified in the other studies and thus omitted). The mRNA levels were measured in the Protein Atlas project (Uhlen et al., 2015) and the protein levels in Geiger et al. 2012 (IBAQ and LFQ quantification). (B) Correlation of relative differences in expression of proteins between this study and other studies (see above). (C) Signal for intracellular drug concentration at 48 hr after treatment is shown for the different drug perturbation conditions and control samples. Adjusted ^∗∗∗^p < 0.001. (D) Correlation between the signal of intracellular drug metabolites across all samples. The measurements from samples treated with GW3965 or atorvastatin are shown as triangle respectively, other conditions are shown as circles. (E) Correlation between HMG-CoA and either atorvastatin, HMGCS1, or summed HMGCS1 + atorvastatin signal. Signal for all metabolites and proteins were scaled between 0 and 1 and error bars show the SD for three independent biological replicates.

Because of the many conditions tested in this study, we could exclude some hypotheses for this differential response. First, we could exclude that the reduced effect in Huh7 cells was due to a reduced inhibition of the drug target HMGCR. The intracellular atorvastatin concentration in Huh7 cells was higher than in HeLa cells (Figure 5C), and Huh7 cells experienced a strong accumulation of HMG-CoA, showing that HMGCR was efficiently inhibited (Figure 4F). Second, we could exclude that SREBP2 does not regulate the expression of these proteins in Huh7 cells because treatment with atorvastatin in full medium led to a significant increase of HMGCS1 abundance (Figure 3B), and knock down of SREBP2 led to a significant reduction of HMGCS1 (Figure 3E). For the other enzymes (ACLY, ACAT2, MVD, IDI1, and FDPS) there was a similar effect that just did not reach significance. Third, we could exclude that SREBP2 is not activated in Huh7 cells in this condition because the other SREBP2 targets in the later sterol committed part of the pathway (FDFT1, LSS, NSDHL, DHCR7, and DHCR24) showed significantly increased abundance (Figure 3B). Hence, it seems that a so far unknown factor influences the expression levels of these enzymes in Huh7 cells.

### Characterization of Other Pathways Affected by Perturbation of Cholesterol Regulation

Among the 16 most often differentially expressed proteins (Figure 2B), 8 proteins belonged to the cholesterol synthesis pathway (ACAT2, FDPS, ACLY, HMGCS1, IDI1, LSS, NSDHL, and FDFT1), confirming that this is the main affected cellular pathway. The remaining eight proteins included two enzymes involved in fatty acid synthesis (FASN and ACSL3), an enzyme of the pyruvate dehydrogenase complex (DLAT), three proteins with functions in protein translation (RPL14, YARS, and GARS), and two enzymes mediating serine biosynthesis (PHGDH and PSAT1). The expression of FASN and ACSL3 primarily increased upon treatment with the LXR agonists (Figure 2B), and coincided with an increased abundance of several fatty acids (Figure S2B). The regulation of the key metabolic enzyme DLAT by LXR agonists and sterol depletion was specific to Huh7 cells.

Perturbation of lipid or cholesterol homeostasis has previously been correlated with changes in expression of genes involved in ER stress and protein synthesis (Fu et al., 2011, Iskar et al., 2013). Supporting this link, RPL14, YARS, and GARS all show a consistently reduced expression in several conditions in HEK, HeLa, and Huh7 cells (Figure 2B). Furthermore, all nine proteins from the chaperonin complex TriC/CCT showed decreased expression, whereas the levels of other chaperones were not consistently decreased (Figure S4). Phosphopeptides of several proteins involved in protein translation (RPL30, RPS3, and EIF5B) or from chaperones (HSP90AB1 and HSPD1) were affected in their abundance by sterol depletion (Table S3; Figure S3). Hence, our data confirm a link between cholesterol and proteostasis-related processes and implicate several proteins that are regulated upon cholesterol depletion.

PHGDH and PSAT1 belong to the serine biosynthesis pathway, and all three enzymes (PHGDH, PSAT1, and PSPH) of this pathway showed a significant reduction in protein levels upon cholesterol depletion with LPDS and atorvastatin in all cell lines except in HepG2 (Figure S5). Concomitantly, α-ketoglutarate, a product of the enzyme PSAT1, showed significantly reduced abundance in HeLa and Huh7 cells but not in HepG2 cells (Figure S5). This coordinated reduction in the abundance of all three enzymes of this pathway and a key cellular metabolite suggests a so far unknown link between statin treatment, serine biosynthesis, and α-ketoglutarate. As the serine biosynthesis pathway is increasingly recognized as a crucial pathway involved in cancer progression (Mattaini et al., 2016), this link might be of high clinical relevance.

### Heterogeneity in Cellular Drug Response, Basal Protein Expression, and Drug Uptake

Despite the fact that the core regulatory processes of cholesterol homeostasis were qualitatively conserved across all tested cell lines, a substantial quantitative variability in the magnitude of the regulation was observed. Therefore, we first assessed the origin of this heterogeneity and then identified the underlying factors with an integrative modeling approach.

The clear clustering of the samples from the same cell line (Figures 2A and S1) confirmed that variable basal expression is an important characteristic of cell lines. To assess if this differential basal expression is truly cell-line-specific, and not the result of lab-specific environmental or technical issues (i.e., different cultivation media, MS approach, or bioinformatic analysis workflow), we compared our data with mRNA and proteomic data from other groups (Geiger et al., 2012, Uhlen et al., 2015). A significant positive correlation was observed between the relative abundances of all proteins (Figure 5B) (transcriptome [R = 0.48], LFQ proteome intensity [R = 0.64]; both p < 2.2 × 10^−16^) and of several key enzymes (Figure 5A). However, the basal abundance levels of the cholesterol synthesis enzymes (HMGCS1, FDFT1, and FDPS) were not generally high or low in any cell line (Figure 5A), suggesting that the variable basal expression was not just the result of variable activity of SREBP2. Despite the different basal levels, the regulation of expression elicited by the perturbations was remarkably similar across proteins of the same pathway and cell line (Figure 2B). Hence, the basal protein levels did not seem to be a major factor determining the variable drug response.

Variation in drug uptake, metabolism, and excretion are recognized to be important parameters affecting intracellular drug concentration and drug response (Meyer et al., 2013). In our study, two different concentrations of each drug were used (see the STAR Methods) and after 48 hr the intracellular drug concentration for atorvastatin, T0901317, GW3965, hydroxy-atorvastatin, atorvastatin lactone, and hydroxy-GW3965 were measured (Table S2). The drug metabolite concentrations correlated well with the intracellular drug concentrations (Figure 5D), and in all cell lines a dose-dependent increase in the intracellular drug concentration was observed, suggesting that drug uptake was not saturated in the tested concentration range (Figure 5C; Table S2). Large differences in the amount of intracellular atorvastatin and GW3965, but not T0901317, were observed between the cell lines, indicating differences in uptake, efflux, or metabolism of these drugs (Figure 5C). Furthermore, the intracellular atorvastatin concentration differed in the liver-derived cell lines (Huh7 and HepG2), but not in HeLa cells, depending on whether cells were grown in medium with fetal bovine serum or LPDS (Figure 5C). But despite analyzing well-studied model cell lines, the observed differences in drug uptake or metabolism could neither be readily explained by the existing literature or the acquired data. The known drug transporters (OATP1B1/3 and MDR1), and metabolizing enzymes (CYP3A4) (Neve et al., 2013, Vildhede et al., 2014) affecting intracellular atorvastatin levels, have been reported to be expressed at very low amounts in our cell lines (Ahlin et al., 2009, Neve et al., 2013), and were also not detected by our MS-based approach. Furthermore, to our knowledge, no sterol-dependent regulation mechanisms affecting atorvastatin uptake or transporters facilitating GW3965 uptake have been described so far.

### Different Factors Affect Accumulation of HMG-CoA

HMG-CoA, the substrate of HMGCR, accumulated upon HMGCR inhibition by statins (Figure 3C). The treatment with a five-times higher atorvastatin concentration typically resulted in the same or slightly higher accumulation of HMG-CoA compared to the treatment of the same cell line with a lower atorvastatin concentration (Figure 4F). However, atorvastatin treatment of cells cultivated in LPDS medium resulted in a significantly higher accumulation of HMG-CoA than if the cells were cultivated in full medium (Figure 4F) (Hela, p < 0.0006; Huh7, p < 0.03; HepG2, p < 8.9 × 10^−5^; nested ANOVA for both drug concentrations), and this was especially prominent in HeLa cells that showed an over 2-fold higher accumulation of HMG-CoA despite similar intracellular atorvastatin concentrations (Table S2; Figure 5C). The possible explanation is that LPDS treatment resulted in increased HMGCS1 levels that lead to more HMG-CoA being produced, and thus a higher accumulation of HMG-CoA upon HMGCR inhibition (Tables S1 and S2; Figures 4E and 4F). To assess how well HMG-CoA levels could be predicted based on the atorvastatin and HMGCS1 levels, we plotted the data points for all conditions and cell lines and assessed the correlation (Figure 5E). Both the intracellular atorvastatin concentration and the HMGCS1 expression correlated significantly with the HMG-CoA levels and a linear combination of HMGCS1 and atorvastatin levels predicted the HMG-CoA levels best (R^2^ = 0.61). Nevertheless, a linear function could not accurately explain the HMG-CoA levels, and more sophisticated models were required to explain the variable drug response. This example highlights the complexity of understanding the variability in drug response, as even the regulation of a molecule so close to the drug target is not only determined by the intracellular drug concentration, but by pharmacodynamic factors such as the turnover rate of HMG-CoA.

### Generating a Network Model Explaining Cellular Cholesterol Regulation

**Figure 6:**
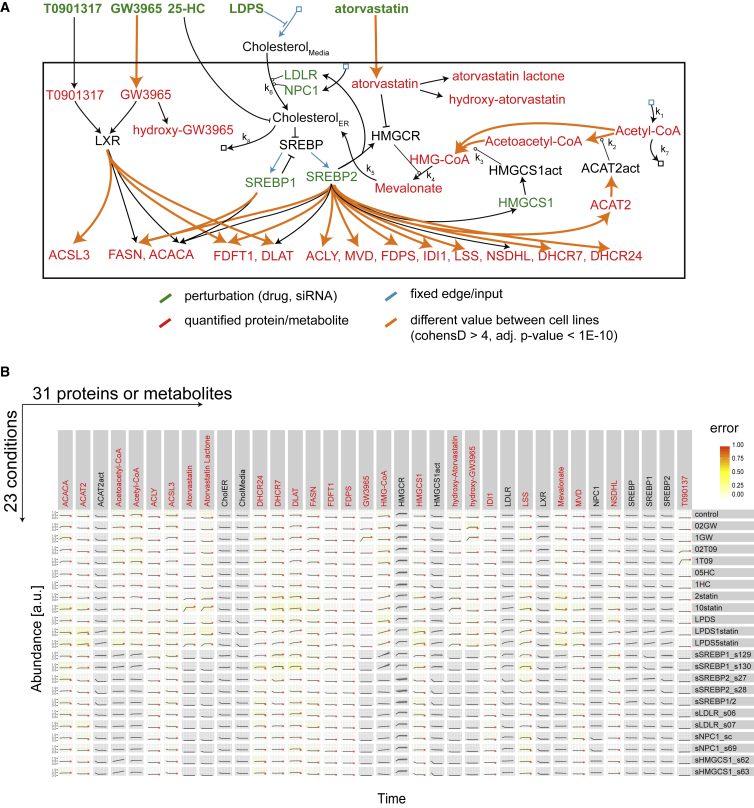
Logic-Based ODE Model of Cellular Cholesterol Regulation (A) Model for cellular cholesterol regulation indicating positive (arrows) and inhibitory (inverted T) interactions between nodes (proteins, metabolites, or protein activities). Metabolic reactions are labeled with k1-7. Edges that show a significant different strength between cell lines are depicted in orange (for further details, see the STAR Methods; see also Figure S6). (B) Overview plot showing the 100 trained models for Huh7 cells (shaded area show the 0.1, 0.25, 0.75, and 0.9 quantile prediction) and the experimental result (red dot indicating quantified signal in the molecules labeled in red font).

An important aim of systems pharmacology is to generate mathematical models that can recapitulate the complex regulatory processes underlying the drug response. Other models exist describing cholesterol regulation and statin treatment (Bhattacharya et al., 2014, Mazein et al., 2013, Paalvast et al., 2015, Watterson et al., 2013), but no model so far captured the heterogeneity in the intracellular drug response after so many perturbations in various cell lines, and considering both SREBP and LXR. With our modeling strategy, we pursued two specific goals: first, to generate a core regulation model for each cell line that describes the quantitative experimental results for proteins and metabolites across all conditions; second, to identify with the help of these models the cause of the observed drug-response heterogeneity. A logic-based modeling strategy allowed us to generate dynamic mechanistic models based on a qualitative understanding of the processes, but without having a detailed chemical knowledge about all the underlying mechanisms (Saez-Rodriguez et al., 2015). Within the CellNetOptimizer (CellNOpt) framework, the same prior-knowledge network was trained with the experimental data from different cell lines to obtain cell-line-specific models (Terfve et al., 2012). Because of the requirement for a prior-knowledge network, the modeling was performed only for the cellular processes with known links to the core regulation model (Figure 1C) (see the STAR Methods). The prior-knowledge network contained the main transcription factors SREBP and LXR, the SREBP feedback interaction, 11 out of the 16 most frequently regulated proteins (Figure 2B), the measured intracellular drug metabolites, and the metabolites from acetyl-CoA to mevalonate of the cholesterol synthesis pathway (Figure 6A). In total, the 21 proteins and 11 metabolites were represented as nodes in the model (Figure 6A). Some metabolites were described by two nodes to account for their intra- and extracellular concentration, and two enzymes (ACAT2 and HMGCS1) were described by an additional node representing their activity. The 44 edges that connected these nodes contained the quantitative description of the interactions (Figure 6A) cast as a set of ordinary differential equations (ODEs) (Terfve et al., 2012). Notably, these ODEs give an overall quantitative description of the functional interaction, rather than describing the biochemical processes with actual chemical constants. To model regulatory interactions, the strength of each edge was described by a Hill-type function. To model the metabolic reactions in the cholesterol synthesis pathway, mass action or Michaelis-Menten functions were employed (see the STAR Methods). The same prior-knowledge network was trained against the bootstrapped experimental data to generate a set of 100 cell-line-specific network models for each cell line (Table S6). The cell-line-specific models explained well (mean root-mean-square error of 0.077) the experimental data for the 25 different measured nodes across the 23 different conditions (Figure 6B).

### Comparing the Different Cell-Line-Specific Models

**Figure 7:**
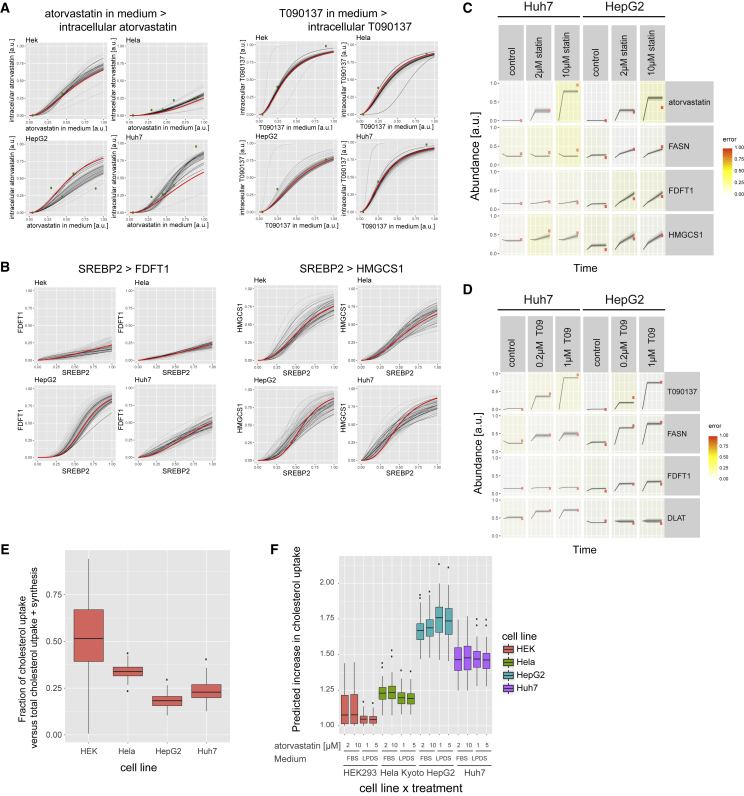
Comparing Cell-Line-Specific Models of Cellular Cholesterol Regulation (A) Transfer function (100 for each cell line) describing which intracellular drug concentration is approximated by the model for a given extracellular drug amount. Green dots indicate the manually defined input value and corresponding quantified intracellular concentration. The red curve depicts the transfer function from the best overall model; black curves show the functions for the 10% best fitting solutions. (B) Representation of transfer functions between SREBP2 and target proteins as explained above. (C and D) Depiction of models (lines) and experimental data (red dots). (E) Predicted fraction of cholesterol uptake versus total cholesterol uptake and synthesis. (F) Predicted increase in cholesterol uptake upon statin treatment.

To assess the origin of the heterogeneity in the drug response between the cell lines, we compared the parameters of the cell-line-specific models (Table S6). These parameters defined, for example, the Hill-type transfer functions that could also be plotted for visual inspection. For instance, the curves show how the same extracellular concentration of atorvastatin resulted in a higher intracellular drug concentration in Huh7 than in HeLa cells (Figure 7A), or how a similar activation of SREBP2 led to a much stronger increase in FDFT1 levels in HepG2 than in the other cells (Figure 7B). Conversely, for T090137 and the effect of SREBP2 on HMGCS1 the curves indicate that the strength of these functional interactions is similar across all cell lines (Figures 7A and 7B). In general, the observed variability in drug response could not be reduced to a single or few variable cellular processes. Rather, it was the consequence of a significant rearrangement of multiple biochemical processes that could be represented as a network in which 19 functional interactions differed strongly between the cell lines (Cohen’s D > 4 and adjusted p < 10^−10^) (Figures 6A and S6; Table S6). These 19 edges predominantly described the processes of drug uptake and the effect of the transcription factors on the protein expression. Other processes were more conserved, such as most reaction rates within the enzymatic pathways, or the activation of the transcription factors by the perturbations.

Importantly, differences in the pharmacodynamics between cell lines may dominate over the differences in pharmacokinetic processes, such as drug uptake or metabolism. For example, the FASN and FDFT1 expression in Huh7 cells reacted less strongly to stimulations of statins and LXR agonists than in HepG2 cells, even though the Huh7 cells experienced a higher intracellular drug concentration (Figures 7C and 7D). Other proteins, such as DLAT and HMGCS1, responded similarly or more pronounced in Huh7 cells, showing that Huh7 cells are not simply less responsive to these drugs. In summary, these results thus highlight the importance of understanding the pharmacodynamic differences in addition to the variability of pharmacokinetic processes between genetically different cells.

## Discussion

We performed a systems pharmacology study of cellular cholesterol regulation using MS-based quantification of drugs, metabolites, and proteins across a panel of four human cell lines. For each cell line, ODE-based models were generated that explained the drug response and captured the variability of intracellular drug concentration (pharmacokinetics) and drug-response phenotypes (pharmacodynamics). Comparison of these models showed that the main regulatory feedback mechanisms were qualitatively conserved, but that, in addition to variability in drug uptake and metabolism (pharmacokinetics), significant variability in the pharmacodynamic response existed. Importantly, the variable drug response could not be explained by one major underlying factor, but emerged from the behavior of the system as a whole.

Even though cholesterol regulation is a well-characterized biological process, new insights were added to the complementation mechanism of SREBP2 for SREBP1, the effect of LXR activation on FDFT1 expression, and the effects of cholesterol depletion on other cellular pathways. Also, drug side effects were captured, such as the increase in lipids upon LXR activation (Hong and Tontonoz, 2014). In addition, hardly any knowledge existed that quantitatively described the observed complex interplay of many variable functional interactions (Figure 6A). The use of quantitative systems pharmacology models has been advocated to capture such complexity (Berg et al., 2010, van Hasselt and van der Graaf, 2015), and this study demonstrates that MS can support such large systems pharmacology studies.

A main advantage of models is the possibility to infer processes that were not directly measured. As an example, our models predict that Huh7 and HepG2 cells obtain a smaller fraction of their required cholesterol from cholesterol uptake than HEK293 and HeLa cells (Figure 7D). This would explain why HEK293 and HeLa cells responded more strongly to impaired cholesterol uptake (LPDS) and Huh7 and HepG2 to the treatments inhibiting cholesterol synthesis (atorvastatin) (Figure 4E). This observation is surprising considering that, in humans, the liver is responsible for 70% of the LDL clearance and only 10% of the total cholesterol synthesis (Dietschy et al., 1993), and suggests that liver-derived cell lines (Huh7 and HepG2) rely more strongly on cholesterol synthesis than *in vivo* hepatocytes and, more generally, that differences between cell lines cannot be exclusively attributed to their tissue of origin. Moreover, our models predict a 63%–73% increase of cholesterol uptake in HepG2 cells upon statin treatment (Figure 7E). In agreement with this, a study reported a ∼60% increase in LDL binding and 1.8- to 2.1-fold higher LDL internalization (Scharnagl et al., 2001). This suggests that our models can predict a medically relevant cellular phenotype. For the other cell lines the predictions still need to be experimentally confirmed.

Personalized models have also been built for genome-scale metabolic networks using a constrained-based approach (Yizhak et al., 2014) or the MASS approach (Bordbar et al., 2015). In contrast to these studies, our approach generates dynamic models based on experimental time course data and the observed differences between the dynamics thus are directly derived from experimental data. By integrating large-scale quantitative data into a framework of mechanisms-based biological knowledge, modeling bridges data-driven and hypothesis-based biology. Whereas the models incorporated the majority of the most frequently differentially expressed proteins, many affected biomolecules could not be incorporated into the models because insufficient knowledge is presently available on how they functionally interact with the core regulation model. This missing knowledge can guide research toward processes that will help explain the full drug-response phenotype once they are mechanistically better understood.

A main goal in personalized medicine is the generation of models that accurately predict the drug response based on measurable biomarkers, such as genotype, protein, or metabolite quantities. In this study, we identified that the main variable processes determining variable drug response were related to drug uptake and to the effects of transcription factors on protein levels (Figure 6A). Therefore, as a next step, we will need to identify biomarkers that determine the strength of these variable functional interactions. For example, mutations in the promotor of SREBP target genes could explain the difference in regulation of the respective protein levels. As the identification of such biomarkers requires an extensive effort and the analysis of many more cell lines, it is crucial to first identify the most variable processes relevant for the variable drug response with an approach as outlined here. Only then can the search for predictive biomarkers be focused on the most relevant functional interactions.

## STAR★Methods

### Key Resources Table

**Table:** 

REAGENT or RESOURCE	SOURCE	IDENTIFIER
**Chemicals, Peptides, and Recombinant Proteins**		

25-Hydroxycholesterol	Cayman Chemical	Cat#11097
Atorvastatin	Cayman Chemical	Cat#10493
GW3965	Sigma	Cat#C6295
Trypsin	Promega	Cat#V5113
LysC	WAKO	Cat#121-05063
Lipofectamine RNAiMAX	ThermoFisher	Cat#13778030
iRT peptides	Biognosys	
DMEM high glucose	GIBCO	Cat#41966
DMEM low glucose	GIBCO	Cat#31885
MEM	GIBCO	Cat#41090
LPDS	PAN Biotech	Cat#P30-3401
TiO2	GL Science Inc	Cat#5020-75000

**Deposited Data**		

Proteomics data	PRIDE	PXD005955
Data and scripts for modeling see Data File S1	Zenodo	https://doi.org/10.5281/zenodo.999543

**Experimental Models: Cell Lines**		

HEK293	ATCC	ATCC Cat#CRL-1573; RRID: CVCL_0045
HeLa Kyoto	Pepperkok lab, EMBL	RRID: CVCL_1922
Huh7	JCRB via Runz Lab, University of Heidelberg	JCRB Cat#JCRB0403; RRID: CVCL_0336
HepG2	ATCC	ATCC Cat#HB-8065; RRID: CVCL_0027

**Oligonucleotides**		

see Table S4 for siRNA seqeuences		

**Software and Algorithms**		

OpenSWATH	Röst et al., 2014	http://www.openswath.org
SWATH2stats	Blattmann et al., 2016	http://bioconductor.org/packages/SWATH2stats/
mapDIA	Teo et al., 2015	https://sourceforge.net/projects/mapdia/
CNORode (see Data File S1)	Zenodo	https://doi.org/10.5281/zenodo.999543
Skyline	MacLean et al., 2010	https://skyline.ms

**Other**		

Combined assay library	Rosenberger et al., 2014	http://www.swathatlas.org/

### Contact for Reagent and Resource Sharing

Further information and requests for resources and reagents should be directed to and will be fulfilled by the Lead Contact Dr. Peter Blattmann (blattmann@imsb.biol.ethz.ch). Contact person for modeling-related questions is Prof. Dr. Julio Saez-Rodriguez (saezrodriguez@combine.rwth-aachen.de).

### Experimental Model and Subject Details

#### Tissue Culture Cell Lines

The different cell lines were obtained from the following sources: HEK293 (ATCC® CRL-1573™) from ATCC; Hela Kyoto were a gift from the Pepperkok lab, EMBL Heidelberg; Huh7 (JCRB0403) were a gift from the Runz lab, University of Heidelberg; HepG2 (ATCC® HB-8065™) from ATCC. All cell lines were authenticated by profiling highly-polymorphic short tandem repeat loci (STRs) and tested for mycoplasma contamination. The sex of the cell lines are as follows; Female: HEK293 and HeLa Kyoto. Male: Huh7, HepG2. The cell lines were cultured in the following media: HEK293 and Huh7 in DMEM high glucose (GIBCO #31885), HeLa Kyoto in DMEM low glucose (GIBCO #31885), HepG2 in MEM (GIBCO #41090). All media contained pyruvate and were supplemented with additional 10% FBS and 2mM L-Glutamine. Cells were grown at 37°C with 5% CO_2_ and saturated air humidity.

### Method Details

#### Tissue Culture

The experiment workflow is depicted in Figure 1A and the experiments were performed in the following manner: Cells were seeded into 6-well dishes at a density that the cells reached 90% confluence when harvested after 2-3 days. For phosphopeptide enrichment a 15 cm dish was used to cultivate the cells. The cells were treated one day after seeding with drugs or siRNAs. For the drug treatment, the drugs were dissolved prior in DMSO (Atorvastatin, GW3965, T090137) or EtOH (25-Hydroxycholesterol) and (0.1% v/v) DMSO and EtOH was added to a separate well as control treatments. Hence, in total three negative controls were performed for the drug perturbations: untreated, 0.1% DMSO treated, and 0.1% EtOH treated cells. Cells treated with LPDS were washed twice with warm phosphate-buffered saline before changing to medium containing 10% LPDS instead of FBS. The cells were harvested 48 h after adding the drug. For the siRNA treatment, SilencerSelect® siRNAs (for sequences see Table S4) were dissolved prior in H_2_O and transfected using Lipofectamine RNAiMax according to the manufacturer protocol to obtain an end concentration of 5 nM. Knockdown of SREBF1 and 2 (sSREBF1/2) was performed by mixing the two siRNAs targeting SREBF1 and the two siRNAs targeting SREBF2 (total end concentration 5nM). As control samples, cells were treated with an siRNA targeting no coding gene (called sNeg9) or cells were mock-transfected with transfection reagent and water instead of siRNA (mock). Hence, three negative controls were performed per experiment: untreated, mock, sNeg9. SiRNA-transfected cells were harvested 72 h post-transfection.

Samples from the same cell line were grown and harvested together (blocking) but the order of harvesting the different wells was changed between different biological replicate to not introduce a possible systematic bias.

All treatments resulted in comparable cell growth except for HEK293 cells treated with 10 μM atorvastatin and HEK cells treated with LPDS + 5 μM atorvastatin. In addition, the HEK293 cells treated with LPDS + 1 μM atorvastatin in 15 cm dishes for phosphoenrichment were detached after 48h. These samples were therefore not harvested.

#### Preparation of Samples

##### Metabolomics

For the metabolomics analysis the cells were directly harvested in the tissue culture dishes. Cells were rapidly washed twice with warm 2 ml of freshly prepared 75mM ammonium carbonate solution pH 7.4, before the culture dish was snap-frozen on liquid nitrogen. Metabolites were extracted three times with 400 μl methanol/acetonitrile/water (2:2:1 v/v) at -20°C and each time incubated for 10 min at -20°C. The three extractions of each sample were pooled, centrifuged at 20’000 g for 10 min to get rid of cell debris, and supernatants were stored at -80°C until MS analysis.

##### Proteomics

Cells were harvested for proteomics measurements by washing once with ice-cold phosphate-buffered saline, scraping off the cells, aspirating the phosphate buffer-saline and freezing the cell pellet in liquid nitrogen. Cells were lysed using 8 M Urea in 100 mM Ammonium Bicarbonate with the help of sonication for 10 min. The lysate was reduced using 2.5 mM tris(2-carboxyethyl)phosphine (TCEP) for 30 min at 37°C and alkylated using 40 mM Iodoacetamide for 45 min at 25°C in the dark. The protein amount was measured using the Bicinchoninic acid (BCA) assay and 60 μg protein was digested with LysC (1:100) for 4 h and Trypsin (1:75) over night. Samples were diluted to 6 M and 1.5 M Urea using 100 mM Ammonium Bicarbonate for digestion with LysC or Trypsin respectively. The digestion was stopped by adding trifluoroacetic acid until a pH∼2-3 was reached. The digested peptides were desalted using C18-columns (The Nest Group Inc.), washed with 2% acetonitrile and 0.1% trifluoroacetic acid in H_2_O, eluted with 50% acetonitrile and 0.1% trifluoroacetic acid in H_2_O and subsequently dried in a speedvac. The dried peptides were dissolved in 2% acetonitrile and 0.1 formic acid in H_2_O and iRT peptides (Biognosys) were added to the sample.

##### Phosphoproteomics

Cells were harvested for proteomics measurements by washing once with ice-cold phosphate-buffered saline, scraping off the cells and freezing the cell pellet in liquid nitrogen. Cells were lysed and digested using 8 M Urea in 100 mM Ammonium Bicarbonate and sonication. The lysate was reduced using 5 mM TCEP for 30 min at 37°C and alkylated using 10 mM Iodoacetamide for 45 min at 25°C in the dark. The protein amount was measured using a BCA Assay and 1mg proteins was used for digestion with LysC (1:150) for 4h and Trypsin (1:75) over night. The digested peptides were purified using C18-columns (Waters), washed with 2% acetonitrile and 0.1% trifluoroacetic acid in H_2_O, eluted with 50% acetonitrile and 0.1% trifluoroacetic acid in H_2_O and subsequently dried in a speedvac. The dried peptides were then dissolved in loading buffer for phosphoenrichment (6% trifluoroacetic acid and 80% ACN in H_2_O) and incubated for 60 min with 1.25 mg TiO_2_ beads (GL Science Inc.). The beads were washed twice with loading buffer, twice with buffer C (80% acetonitrile, 0.1% trifluoroacetic acid in H_2_O), twice with buffer D (50% acetonitrile and 0.1% trifluoroacetic acid in H_2_O) and then twice with buffer E (0.1% trifluoroacetic acid in H_2_O). The phosphopeptides were eluted with 0.3 M ammonium hydroxide pH 10.5 and re-acidified immediately to pH 2-3. Afterwards the phosphopeptides were purified on C18-columns (The Nest Group Inc.) as described before and after drying dissolved in 2% acetonitrile and 0.1% formic acid in H_2_O and iRT peptides (Biognosys) were added to the sample.

#### Mass Spectrometry-based Acquisition of Samples

##### Metabolomics

Untargeted measurements of metabolites was performed by direct injection mass spectrometry using a quadrupole-coupled time of flight instrument (Agilent 6550 Q-TOF) with the following settings: negative mode; 4 GHz high resolution mode; scanning the m/z range from 50 to1000 as previously reported (Fuhrer et al., 2011).

##### Proteomics

The proteomics samples were measured on a Sciex TripleTOF 5600 instrument (Sciex, Concord, Canada). The peptides were separated by nano-flow liquid chromatography (NanoLC Ultra 2D, Eksigent) with a flow of 300 nl/min using a NanoSpray III source with a heated interface (Sciex, Concord, Canada). A fused silica PicoTip™ Emitter (inner diameter 75 μm) (New Objective, Woburn, USA) manually packed with 21 cm C18 beads (MAGIC, 3 μm, 200 Å, Michrom BioResources, Auburn, USA) was used to separate about 1 μg of peptides along a linear 120 min gradient from 2% to 35% Buffer B (98% acetonitrile and 0.1% formic acid in H_2_O) in Buffer A (2% acetonitrile and 0.1% formic acid in H_2_O). The TripleTOF 5600 was operated in positive ion, high sensitivity SWATH-mode using 64 variable windows between 400 and 1200 m/z (1 m/z overlap). The collision energy was calculated based on a formula for peptides with charge 2+ adding a spread of 15eV. An accumulation time of 250 ms for precursor ions and 50 ms for all fragment ion scans was used that resulted in a total cycle time of about 3.5 s. Samples from the same cell line and biological replicate were injected together in a block design, but randomized within each block. The injection order of the cell line blocks was also randomized.

##### Phosphoproteomics

Phopshopeptide samples were acquired as described above on a Sciex TripleTOF 5600 instrument.

To generate a phospho SWATH-library the samples were in addition acquired in high resolution data-dependent mode. For this control samples and treated samples were pooled and injected twice for each cell line. The MS1 spectra were acquired for 250 ms in the range of 360 to 1460 m/z. The 20 most intense precursors with charge states between 2 and 5 were selected for fragmentation and excluded for reselection for 15 s. The MS2 spectra were acquired for 100 ms in the range of 50 to 2000 m/z.

### Quantification and Statistical Analysis

#### Obtaining Quantitative Measurements from the Mass Spectrometry Data

##### Metabolomics

Ions were annotated to metabolites based on exact mass considering [M-H+] and [M+F-] ions using the metabolite reference list compiled from the HMDB (Wishart et al., 2013). Ions were assigned to metabolites allowing a mass tolerance of 0.001 Da and an intensity cutoff of 1,500 counts as previously described (Fuhrer et al., 2011). The annotation was filtered in three steps: for each ion, only metabolites with the top annotation score were retained; for each metabolite, only the annotation with the top score was retained; and the annotations with adducts such as NaCl, H/Na, H/K were removed. Negative ions for the drugs not present in the database (T0901317: 480.031699 Da, GW3965: 580.1863299 Da, hydroxyl-GW3965: 596.181546 Da, 2(oder 4)-hydroxy-atorvastatin: 573.240091 Da, atorvastatin lactone: 539.234611 Da) were manually added to the reference list for annotation.

##### Proteomics

The data was analyzed using a pipeline configured on the iPortal platform in the lab (Kunszt et al., 2015). The raw SWATH wiff files were converted using ProteoWizard (version 3.0.5533) to profile mzXML files (Kessner et al., 2008). The extraction of the data was performed using the OpenSWATH workflow (Röst et al., 2014) and the combined human assay library (Rosenberger et al., 2014). An m/z fragment ion extraction window of 0.05 Th, an RT extraction window of 600 s and a set of 10 different scores were used. To match features between runs, detected features were aligned using an spline regression with a target assay FDR of 0.01 (Röst et al., 2016). The aligned peaks were allowed to be within 3 standard deviations or 60s after retention time alignment. For runs where no peak was identified the area was requantified using the single shortest Path method (Röst et al., 2016). The data was then processed using the R/Bioconductor package SWATH2stats (Blattmann et al., 2016). Precursors had to pass an m-score threshold of 1E-05 in at least 20% of the 291 files to be selected for further analysis. These threshold resulted in an estimated precursor FDR of 0.0025, peptide FDR of 0.002745 and protein FDR of 0.0140 (using an estimated fraction of false targets (FFT) or π_0_-value of 0.6 for estimating the FDR). In total 24’266 peptides and 111 decoy peptides passed this stringent threshold. Subsequently, only proteotypic peptides and the 7 peptides with the highest signal per protein were selected for quantitative analysis. This resulted in a data matrix containing 4.5 10^6^ peak group intensities, from which 78% of peak groups had an m-score of < 0.01. The data was normalized using a local total intensity normalization within a retention time window of 10 min and analyzed for differential expression using mapDIA v1.2.1 (Teo et al., 2015). Differential expression was tested using an independent study design with the settings of selecting a minimum correlation of 0.25, a standard deviation factor of 2, between 3-5 fragments per peptide and at least one peptide per protein.

##### Phosphoproteomics

In total 16 different data-dependent files were used to create a common phospholibrary for the different cell lines. For this the data was searched using XTandem, OMssa and Comet using a Parent mass error of 50 ppm, a fragment mass error of 0.04 Da, 1 missed cleavage was allowed and as modification carbamidomethyl on cystein as a static and phosphorylation on serine, threonine and tyrosine and oxidation on methionine as a variable modification was added. A 0.01 iprophet-peptide FDR cutoff was used to control for false identifications. For each annotated spectra a false localization score was calculated using Luciphor2 (Fermin et al., 2013) and annotations with a false localization rate (FLR) of lower than 0.01 are annotated as localized phosphopeptides (localized modification is depicted in Protein Name, see Table S3). Phosphopeptide that showed a FLR above 0.01 were viewed as non-localized and contain no localized modification in the protein name label, but are labelled “_Phospho_1”. A SWATH assay spectral library was generated as described before using a distance of 2 min to separate adjacent peaks and the TPP (Schubert et al., 2015). Adjacent peaks are labelled with “Subgroup” (see Table S3). This resulted in a SWATH-assay library containing 5275 different phosphopeptides and proteotypic phosphopeptides mapping exclusively to 1978 different Swissprot protein identifiers.

The extraction of the data was performed using the OpenSWATH workflow (Röst et al., 2014) as described above. The data was then processed using the R/Bioconductor package SWATH2stats (Blattmann et al., 2016). Precursors had to pass an m-score threshold of 0.01 in 3 biological replicates of one condition to be selected for further analysis. These threshold resulted in an estimated precursor and peptide FDR of 0.0179 (using an estimated fraction of false targets (FFT) or π_0_-value of 0.45 for estimating the FDR). In total 2209 peptides and 88 decoy peptides passed this stringent threshold. This resulted in a data matrix containing 1.0 10^5^ peak group intensities, from which 61% of peak groups had an m-score of < 0.01. The data was then normalized using a total intensity normalization and analyzed for differential expression using mapDIA v2.4.1 (Teo et al., 2015). Differential expression was tested using an independent study design with the settings of selecting a minimum correlation of 0.1, a standard deviation factor of 2, between 3-5 fragments per peptide and at least one peptide per protein.

#### Post-processing of the Quantitative Data

##### Metabolomics

In total 1038 different metabolites have been measured in the 159 samples (see Table S2). Each sample has been measured twice by mass spectrometry with the exception of one sample: One of the two technical replicate of HeLa cells treated with 5 μM atorvastatin and LPDS was excluded due to lower overall signal. The mean and median CV within the biological replicates was <20% showing that measurements were highly accurate and reproducible. The median CV in the signal across all samples was 54% and within technical injections 4.6%. To assess the differential abundance of each metabolite, in total 50’818 fold changes were calculated for different metabolite and cell line combinations in comparison to the metabolite concentration of the various control conditions of the same cell line. For the drugs dissolved in either EtOH or DMSO the fold change compared to EtOH or DMSO treated cells was calculated. Statistical test was performed using a hierarchical/nested ANOVA for the 3 biological and 2 technical replicates and the p-value was adjusted using the method of Benjamin-Hochberg to obtain FDR estimates. The change in abundance was considered significant if the FDR was below 0.01 and both the median and mean log2FC was more than ±0.5 against both the untreated samples and to DMSO or EtOH sample if applicable. Using the analysis and thresholds outlined above, 1397 (2.7%) comparisons from 435 (42%) metabolites passed these criteria.

To assess the false discovery rate (FDR) with a different approach we analyzed how many metabolites were regulated if comparing the EtOH and DMSO treated samples to the untreated sample. Assuming that EtOH and DMSO did not affect any metabolites, we could estimate an FDR. Hence, the same log2FC and FDR threshold was applied and 24 (0.2%) out of 8’406 tested comparisons corresponding to 21 (2.0%) out of the 1038 metabolites passed this significance threshold. This shows that the threshold is stringent and the false discovery rate is indeed much lower than 1% on the level of the comparisons and on the metabolite level was around 2%. However, for the analysis of most conditions (all except LPDS), the metabolites had to be significantly different in abundance both against the untreated sample and the EtOH or DMSO condition. Thus the estimated FDR is also on metabolite level lower than 2% and probably in the range of 1%.

##### Proteomics

Digested protein samples have been acquired on Sciex TripleTOF 5600. In total 93’294 different transitions from 13’551 proteotypic peptides and 4311 different proteins have been measured (see Table S1). This was performed in 174 samples from 14 different drug treatments and 112 samples from 13 different siRNA treatments performed for each cell line in at least biological duplicates (3 drug treated samples were injected twice). After filtering with SWATH2stats and the correlation threshold in mapDIA, quantitative data for 12’621 different peptides from 3362 proteins were obtained. The median coefficient of variation for the peptide signals was 22% and 11% for the drug perturbations and siRNA perturbations respectively showing that the measurements are highly reproducible. The median coefficient of variation for the protein signals was 18% and 9% for the drug perturbations and siRNA perturbations respectively. The main reason for the lower CV of the siRNA samples was most probably that the measurement were quantified on the same machine within the same month, whereas the drug perturbations have been acquired on different machines over a period of over a year. As a comparison the median CV for the protein measurements across all samples was 38% and 33% for the drug and siRNA experiments and thus substantially higher than the variation in signal between biological replicates. As a threshold for a statistically significant differential expression, the protein abundance had to be changed by at least log2FC of ±0.5 with a FDR < 0.001 against both the untreated and the other control samples (drug treatment: DMSO or EtOH treated cells, siRNA: sNeg9 and mock treated cells). This resulted in the abundance of 694 out of 3362 (21%) proteins being affected by drug treatments and the abundance of 1102 out of 3362 (33%) proteins being affected by at least one siRNA treatment. The overlap consisted of 394 proteins.

##### Phosphoproteomics

In total 17’292 different transitions have been measured and analyzed (see Table S3). This has been performed in 41 samples from in total 7 different conditions per cell line (one drug perturbation measured at 4 different time points and the 48h time point was performed in biological triplicates). After applying a correlation threshold of 0.1 in mapDIA, quantitative data for 2209 different peptides from 1036 proteins were obtained. The median coefficient of variation for the peptide signals was 30% for the phosphopeptides. This was significantly higher than for peptides that were not phosphoenriched which is probably due to the additional phosphoenrichment step. As a threshold for a statistically significant differential abundance, the phosphopeptide had to show at least a log2FC of ±0.5 with a FDR < 0.01 in the 48h time point against the untreated samples. This resulted in the abundance of 525 out of 2063 (25%) phosphopeptides from 349 proteins being affected in at least one cell line. The abundance of 71 peptides from 54 proteins were significantly changed in at least two different cell lines (see Figure S3). As HEK293 cells did not supported the treatment at 48h, this comparison could only be performed for the other three cell lines.

#### Heterogeneity in Basal Protein Expression

To validate the baseline input data for our models, we compared the differences in protein levels in this study to those of other studies that measured mRNA or protein expression in the same cell lines (Geiger et al., 2012, Uhlen et al., 2015). Quantitative estimation of expression were available for HEK293, Hela and HepG2 cells from a proteomic study (Geiger et al., 2012), and the mRNA measurements were used from the Protein Atlas project (Uhlen et al., 2015). Geiger and coworkers estimated the protein abundance with two different approaches (IBAQ and LFQ) and we used both of these estimations for our comparison.

The Table S2 from the Geiger et al. publication (Geiger et al., 2012) was retrieved and the IPI identifiers were converted to HGNC and Ensembl identifiers with R using the BiomaRt (Durinck et al., 2009) R/Bioconductor package. If there were several different abundance levels for the same SwissProt identifier, the value for the entry based on the most peptides was selected. For the proteomics data both the IBAQ values and LFQ values were used. The data from the Protein Atlas was downloaded from the website http://www.proteinatlas.org/about/download (RNA cell line data; accessed 15^th^ September 2015) and the FPKM values were used. The values were then compared to the intensity values obtained from the SWATH data. This comparison was performed only for the 3362 proteins for which data was available for all three cell lines in all three studies. The difference in expression was calculated by subtracting the signal for this protein in a certain cell line from the mean signal for all cell lines. This difference was then compared to the value obtained for the same protein in the same cell line in the other study. The correlation was calculated using Pearson’s product moment correlation and plotted in a scatter plot.

The relative differences in abundance for the key regulators FDFT1, HMGCS1, and FASN from our dataset were in good agreement with the relative abundance determined in the other studies (Figure 5A). Both in our and the other studies, HMGCS1 showed the highest expression in HEK293 cells and FDFT1 was expressed highest in the HepG2 cells. In contrast, the comparison of the FASN abundance did not yield an entirely consistent result across all studies and our data agreed with the IBAQ intensity values from the proteomic study by Geiger et al. (Figure 5A). In the systematic analysis, we compared if the relative differences in the protein levels were similar across the different studies, i.e. if the lower expression of HMGCS1 in our data relative to the other cell lines was also observed in the other studies. This analysis resulted in a significant positive correlation with both studies (R = 0.48;p<2.2 e-16 for the transcriptome, R = 0.64;p<2.2 e-16, for the LFQ intensities of the proteome), demonstrating that the differences in basal expression are a conserved feature of cell lines (Figure 5B). We therefore decided to incorporate the information about the basal input levels into our models.

#### Building Core Regulation and Prior-knowledge Network Model

The core regulation model (Figure 1C) was built based on the known functional interactions of the literature (Brown and Goldstein, 2009). This was also the basis for the prior knowledge network model (Table S5). The transcription factor SREBP was represented as three nodes: SREBP, SREBP1, and SREBP2 with SREBP activating SREBP1 and SREBP2 with an edge described by parameters that were fixed. As the exact underlying mechanism for the complementation of SREBP2 for SREBP1 knockdown was unclear, this functional interaction was depicted with a hypothetical inhibitory edge from SREBP1 to SREBP, the simplest functional interaction that would explain the observed behavior. The edges from the transcription factor to the downstream edges were defined based on previous findings or based on an observed changed abundance after activating either SREBPs or LXR.

#### Logic Modeling Using CNORode

R scripts were used to process the quantitative mass spectrometry data for modeling. The data was scaled between 0 and 1 in the total range of the observed signal across all cell lines in order to keep the relative differences in the signal between the cell lines. The signal from drugs and drug metabolites was scaled between the minimal and maximal recorded value to remove any signal offset. The signal from endogenous metabolites was scaled between 0 and the maximal recorded signal to prevent that small variation in steady-state levels would suddenly show up as big variation in signal. The data was saved in the MIDAS format (Saez-Rodriguez et al., 2008). The R/Bioconductor package CNORode was used. This package converts logic interactions into differential equations (Terfve et al., 2012), and was adapted to add edges with mass action kinetics for metabolic reactions and have a default starting level of 0.5 for most nodes. The following ordinary differential equations were used to describe the metabolic reactions:

$$\begin{array}{l} \frac{d\left[\text{Acetyl}-\text{CoA}\right]}{dt}=\left(0.5\cdot k_{1}-k_{2}\cdot \left[\text{ACAT}2\text{act}\right]\cdot {\left[\text{Acetyl}-\text{CoA}\right]}^{2}-k_{3}\cdot \left[\text{HMGCS}1\text{act}\right]\cdot \left[\text{Acetyl}-\text{CoA}\right]\cdot \left[\text{Acetoacetyl}-\text{CoA}\right]-k_{7}\cdot \left[\text{Acetyl}-\text{CoA}\right]\right)\cdot \tau_{\text{Acetyl}-\text{CoA}}\\ \frac{d\left[\text{Acetoacetyl}-\text{CoA}\right]}{dt}=\left(k_{2}\cdot \left[\text{ACAT}2\text{act}\right]\cdot {\left[\text{Acetyl}-\text{CoA}\right]}^{2}-k_{3}\cdot \left[\text{HMGCS}1\text{act}\right]\cdot \left[\text{Acetyl}-\text{CoA}\right]\cdot \left[\text{Acetoacetyl}-\text{CoA}\right]\right)\cdot \tau_{\text{Acetoacetyl}-\text{CoA}}\\ \frac{d\left[\text{HMG}-\text{CoA}\right]}{dt}=\left(k_{3}\cdot \left[\text{HMGCS}1\text{act}\right]\cdot \left[\text{Acetyl}-\text{CoA}\right]\cdot \left[\text{Acetoacetyl}-\text{CoA}\right]-\frac{k_{4}\cdot \left[\text{HMGCR}\right]\cdot \left[\text{HMG}-\text{CoA}\right]}{kM_{4}+\left[\text{HMG}-\text{CoA}\right]+\frac{kM_{4}+\left[\text{atorvastatin}\right]}{kI_{\text{atorvastatin}}}}\right)\cdot \tau_{\text{HMG}-\text{CoA}}\\ \frac{d\left[\text{Mevalonate}\right]}{dt}=\left(\frac{k_{4}\cdot \left[\text{HMGCR}\right]\cdot \left[\text{HMG}-\text{CoA}\right]}{kM_{4}+\left[\text{HMG}-\text{CoA}\right]+\frac{kM_{4}+\left[\text{atorvastatin}\right]}{kI_{\text{atorvastatin}}}}-k_{5}\cdot \left[\text{Mevalonate}\right]\right)\cdot \tau_{\text{Mevaloante}}\\ \frac{d\left[\text{CholER}\right]}{dt}=\left(k_{6}\cdot \left[\text{CholMedia}\right]\cdot \left[\text{LDLR}\right]\cdot \left[\text{NPC}1\right]+\;k_{5}\cdot \left[\text{Mevalonate}\right]\cdot -k_{8}\left[\text{CholER}\right]\right)\cdot \tau_{\text{CholER}} \end{array}$$

Some edges and input were fixed in order to reduce the number of parameters that need to be estimated and are represented in blue in Figure 6A. The objective function to facilitate finding an optimal solution was adapted from the original package: First, nodes that were not quantified (CholER, CholMedia, LDLR and NPC1) were penalized if they deviated in the untreated condition from 0.5 along the time simulated. Second, measured nodes received a higher weight if they showed differential abundance. Third, three data points in between the last time point (48h or 72h) and the baseline/control data were introduced by estimating the level to be 0.5, 0.8 or 0.9 of the total difference in abundance. These additional data points, that received lower weight than the measured data points, were introduced to prevent any biologically unexpected oscillatory behavior. The same network and bounds for all parameters were used to train the network for the different cell lines. For each cell line 100 different trainings were performed and for each training > 50’000 different parameter sets were tested using the adapted CNORode and MEIGO package (Egea et al., 2014, Terfve et al., 2012). The prior knowledge network was trained against the bootstrapped data for each cell line. Bootstrapping from the in total 11’572 different data-points was performed by subsampling from the different biological replicates. See Data File S1 for script and the R packages used in the modeling approach.

To assess which edges are different the parameters were compared between the different cell lines. Parameters that had a Cohen’s D effect size larger than 4 and an adjusted p-value < 1e-10 from a Kruskall-Wallis test were deemed significantly different.

#### Generation of Heatmaps

The heatmaps depicting the abundance (Figures 2A and S1) were generated from the data matrix containing the quantitative values which have been scaled to 1 for the maximal value for each analyte (protein or metabolite) across all measured samples (data from biological replicates was averaged). The clustering was performed using the ward.D2 algorithm and the figures were generated using R function heatmap.2 from the gplots package. Treatments were abbreviated as followed: LPDSstatin: LPDS + atorvastatin, T09: T090137, GW: GW3965, HC: 25-Hydroxycholesterol. The numbers indicate the different concentration in μM, except for 25-hydroxycholesterol the concentration is in μg/ml.

The heatmaps depicting the relative change in abundance (Figures 2B, 3B–3E, and S2) show the log2FC in abundance between the indicated condition and the abundance of the untreated cells of the same cell line. See STAR Methods above for how the log2FC was calculated. Arrows indicate a significant change in expression (|log2FC| > 0.5 and FDR < 0.001 (Proteins) FDR < 0.01 (Metabolites)) against both the untreated cells and the respective control treated cells (DMSO or EtOH for the drug dissolved in either solvent, sNeg9 and mock infected cells for siRNA treatments). The log2FC for the different drug concentrations is depicted in the splitted cells in the following order (control: DMSO, EtOH, GW3965: 0.2 μM, 1 μM, T090137: 0.2 μM, 1 μM, 25HC: 0.5 μg/ml, 1 μg/ml, atorvastatin: 2 μM, 10 μM, LPDS+atorvastatin: 1 μM, 5 μM). Grey values are shown if the samples were excluded due to inhibition of cell growth (e.g. HEK293 cells treated with LPDS + 5μM atorvastatin and 10μM atorvastatin). The log2FC for the different siRNAs targeting the same genes are depicted in the following order (control: mock, sNeg9, sSREBF1: s129, s130, sSREBF2: s27, s28, sLDLR: s06, s07, sNPC1: c, s69, sHMGCS1: s62, s63). LPDS and sSREBF1/2 treated samples do not have splitted cells.

### Data and Software Availability

The mass spectrometry proteomics data have been deposited to the ProteomeXchange Consortium via the PRIDE partner (Vizcaino et al., 2016) repository with the dataset identifier ProteomeXchange: PXD005955.

The scripts for modeling and to reproduce the figures from the modeling (Data File S1) are also deposited on Zenodo (https://doi.org/10.5281/zenodo.999543).

OpenSWATH (Röst et al., 2014), SWATH2stats (Blattmann et al., 2016), mapDIA (Teo et al., 2015) and the combined assay library (Rosenberger et al., 2014) have all been published and deposited in the respective journals. Furthermore, the OpenSWATH related software is available on http://www.openswath.org.

## Author Contributions

P.B., J.S.-R., and R.A. conceived and designed the project. P.B. and F.F. performed the experiments. M.Z. acquired and analyzed the metabolomic data. P.B. acquired and analyzed the proteomic data, generated the figures. P.B. and D.H. programmed the software and performed the model fit and analysis, advised by J.S.-R., P.B., and R.A. wrote the original draft. All co-authors contributed in reviewing and editing the manuscript. P.B., U.S., J.S.-R., and R.A. provided funding and resources to support the project.

## Supplemental Information

Document S1. Figures S1–S6

Table S1. Protein Results, Related to Figure 2 Protein results for all samples. Sample labels consist of a concatenated string describing the cell line, condition, and biological replicate. Estimated difference in abundance between two samples obtained from the software mapDIA (Teo et al., 2015). For further details see the STAR Methods. For raw data and quantitative estimation for peptide and protein abundances see ProteomeXchange: PXD005955.

Table S2. Metabolite Results, Related to Figure 3C Metabolite results for all samples. (1) Raw data after quantification. (2) Data (relative intensity in ppm) after normalization. (3) Results of the statistical analysis comparing the treated versus the control conditions using a nested ANOVA. For further details see the STAR Methods.

Table S3. Phosphoprotein Results, Related to Figure 3D Phosphopeptide results for all samples. Sample labels consist of a concatenated string describing the cell line, condition, time point, and biological replicate. (1) Raw data that were used as input for the analysis. (2) Quantitative estimation for peptide abundances, (3) Estimated difference in abundance between two samples. The software mapDIA (Teo et al., 2015) was used for this analysis. For further details see the STAR Methods.

Table S4. Sequence of siRNAs, Related to the STAR Methods Sequence for Silencer Select siRNAs from Life Technologies used in this project.

Table S5. Prior-Knowledge Network Model, Related to Figure 6 SIF file of the prior-knowledge network used for modeling. See also Data File S1.

Table S6. Model Parameters, Related to Figure 6 Estimated parameters contains the estimated parameters for the different edges. Each row represents one parameter set for a model. In total 100 models for each cell line have been trained from the bootstrapped data. Table comparison of parameters) Results of statistical comparison of the parameters for the models between cell lines. Displayed are the mean values for both cell lines, the p value from a t test and Kruskal-Wallis test, the Cohens D effect size and the Benjamini and Hochberg adjusted p values for both statistical tests.

Data S1. Modeling Scripts, Related to Figure 6 Zipped file containing the scripts and code used for modeling and to produce the figures related to modeling.

Document S2. Article plus Supplemental Information
