# Supplementary material for: Systems Pharmacology Dissection of Cholesterol Regulation Reveals Determinants of Large Pharmacodynamic Variability between Cell Lines
Source: Cell Syst. 2017 Dec 27;5(6):604–619.e7. doi: 10.1016/j.cels.2017.11.002 (PMC5747350; doi:10.1016/j.cels.2017.11.002)
Supplement: Document S1. Figures S1–S6 [file mmc1.pdf]

Cell Systems, Volume *5*

Supplemental Information

Systems Pharmacology Dissection of Cholesterol Regulation Reveals Determinants of Large Pharmacodynamic Variability between Cell Lines

Peter Blattmann, David Henriques, Michael Zimmermann, Fabian Frommelt, Uwe Sauer, Julio Saez-Rodriguez, and Ruedi Aebersold

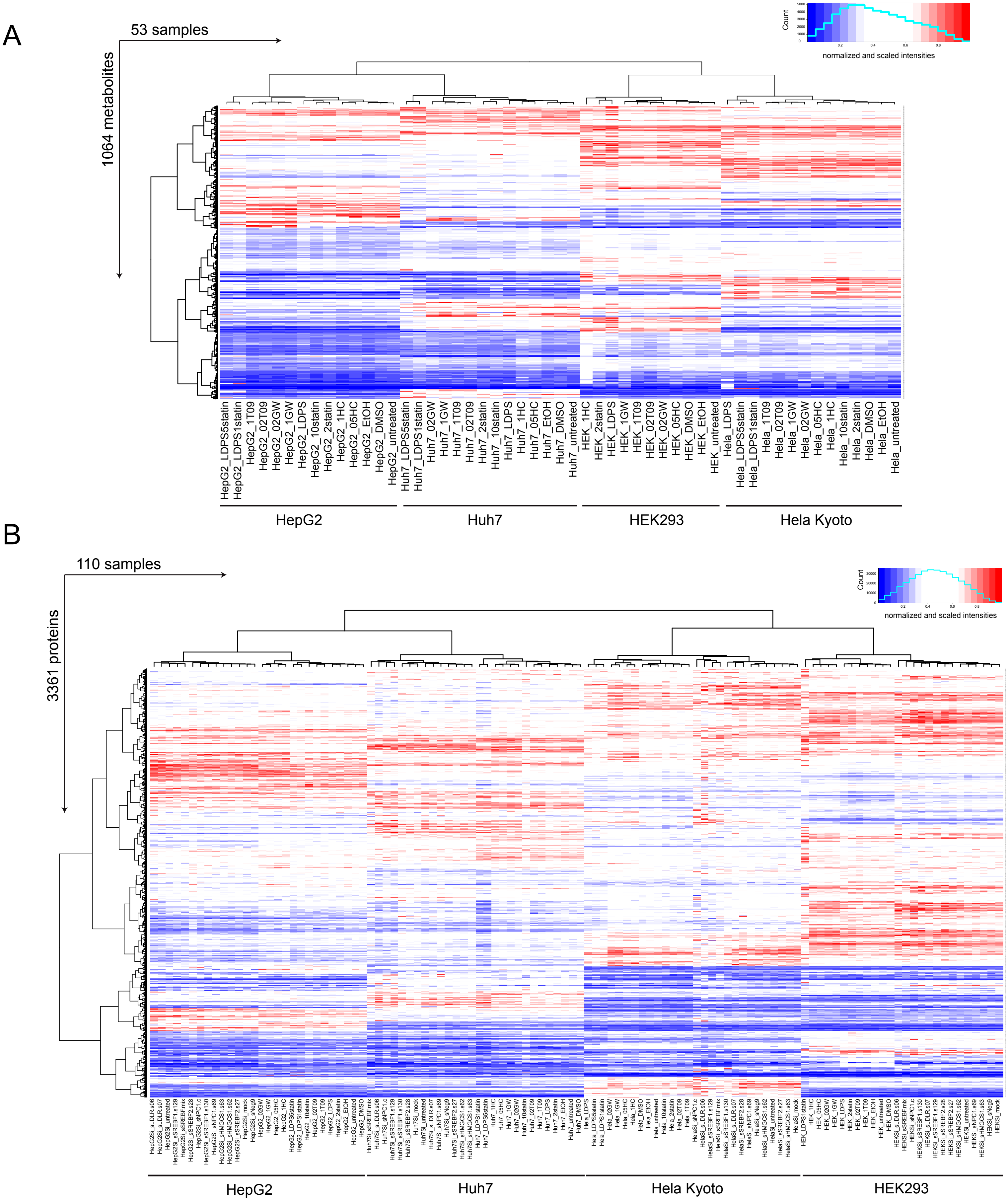

Figure S1: Quantitative results for metabolites and proteins, Related to Figure 2A.

Complete quantitative results for metabolites (A) and proteins (B) for all perturbations. Intensities were normalized and scaled between 0 and 1 for each biological sample (values from biological replicates were averaged). The data was clustered using the wardD2 hierarchical clustering algorithm. Metabolite data do not include drug metabolites. Treatments were abbreviated as followed: LPDSstatin: LPDS + atorvastatin, T09: T090137, GW: GW3965, HC: 25-Hydroxycholesterol. The numbers indicate the different concentration in µM, except for 25-hydroxycholesterol the concentration is in µg/ml. For the siRNA treatments the last digits of the siRNA identifier number (Table S4) is indicated. For further details see Methods.

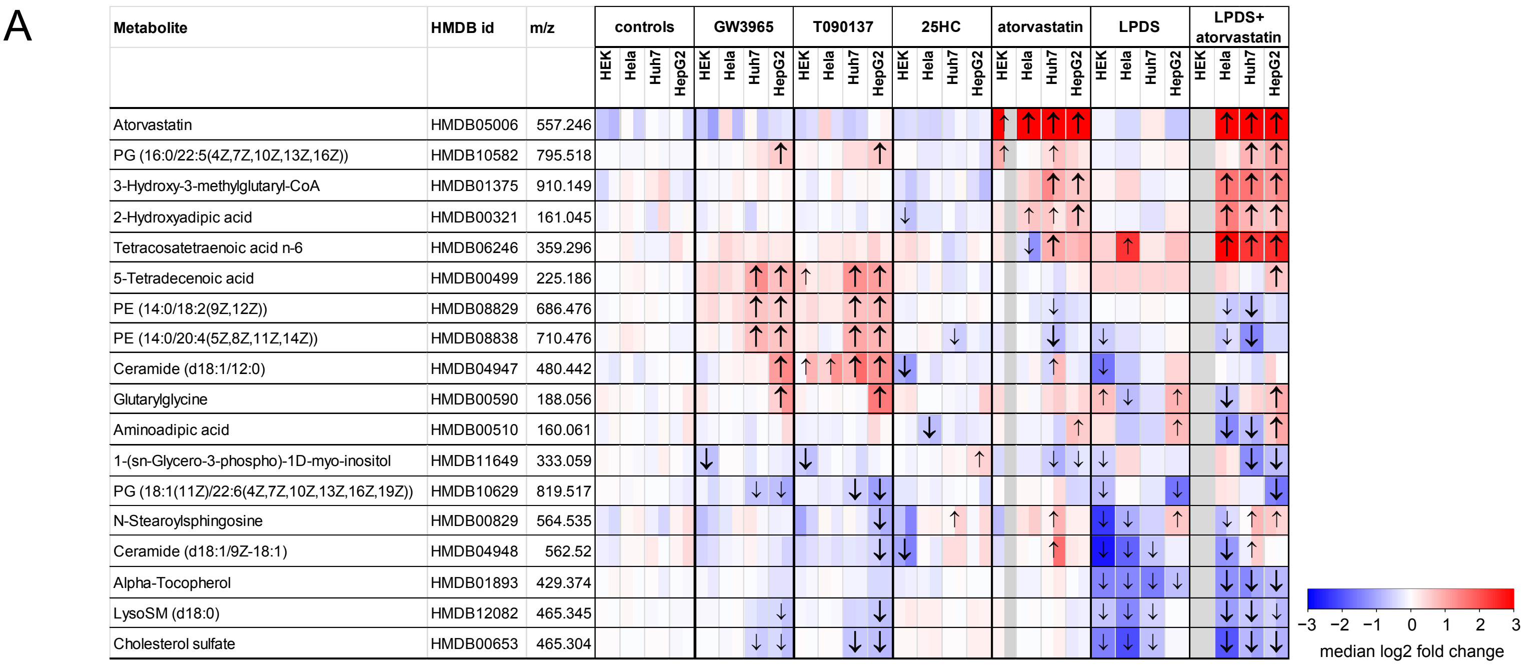

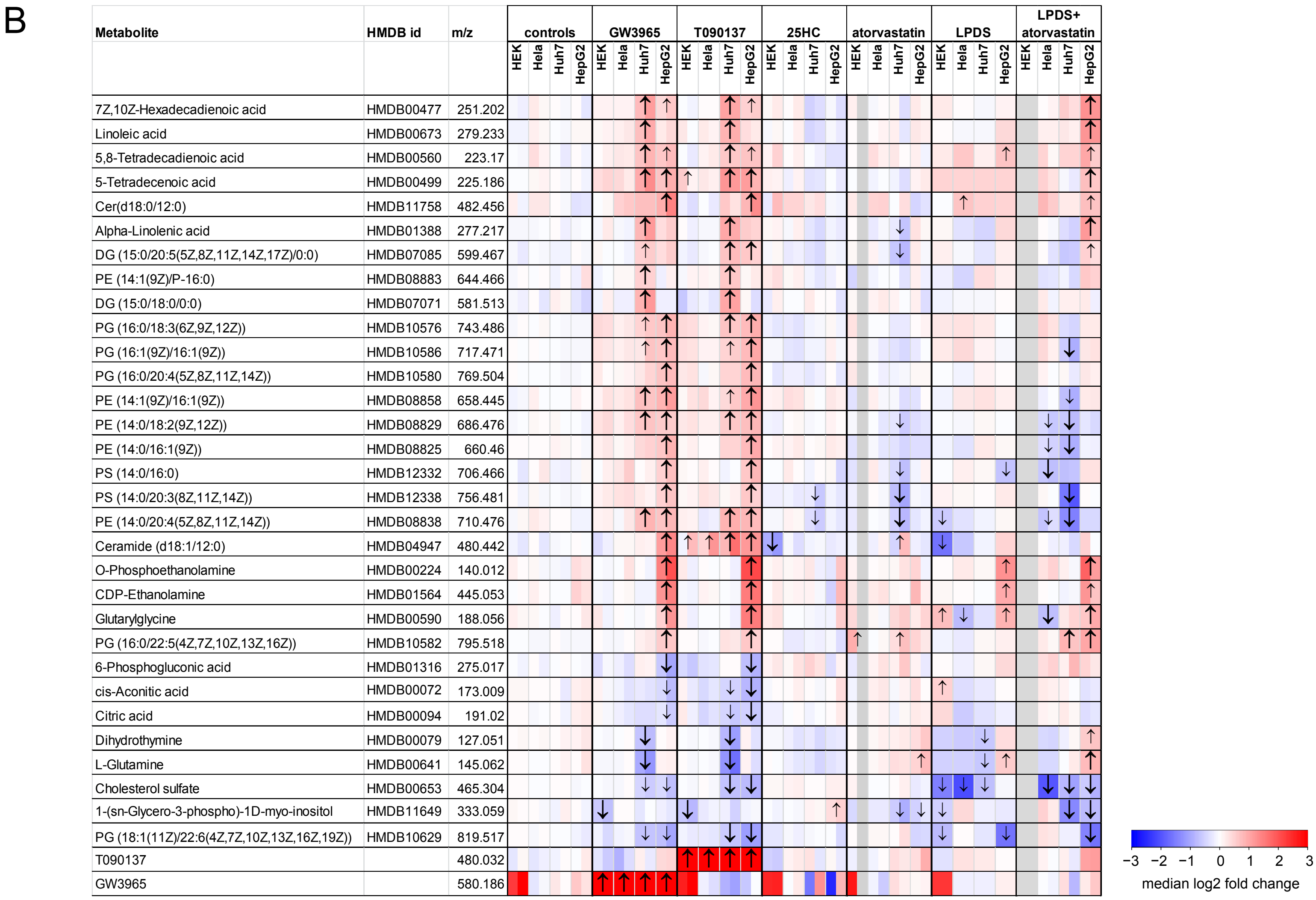

Figure S2: Metabolites with changed abundance, Related to Figure 2B

Shown are metabolites whose abundance was affected in 10 or more conditions (A) or upon at least 4 LXR stimulation conditions (B) (FDR < 0.01, mean and median log2FC > ±0.5 from untreated and DMSO or EtOH perturbed control samples for drugs). The arrows indicate the direction of the significant differential abundance of the metabolite upon treatment with the drug. Arrows in bold indicate a significant change in abundance for both concentrations of the drug. For all conditions except LPDS two different concentrations were used (control: 0.1% DMSO, 0.1% EtOH, GW3965: 0.2µM, 1µM, T090137: 0.2µM, 1µM, 25HC: 0.5 µg/ml, 1 µg/ml, atorvastatin: 2µM, 10µM, LPDS+atorvastatin: 1µM, 5µM). HEK cells treated with LPDS + atorvastatin and 10µM atorvastatin affected cell viability and were excluded from analysis (grey).

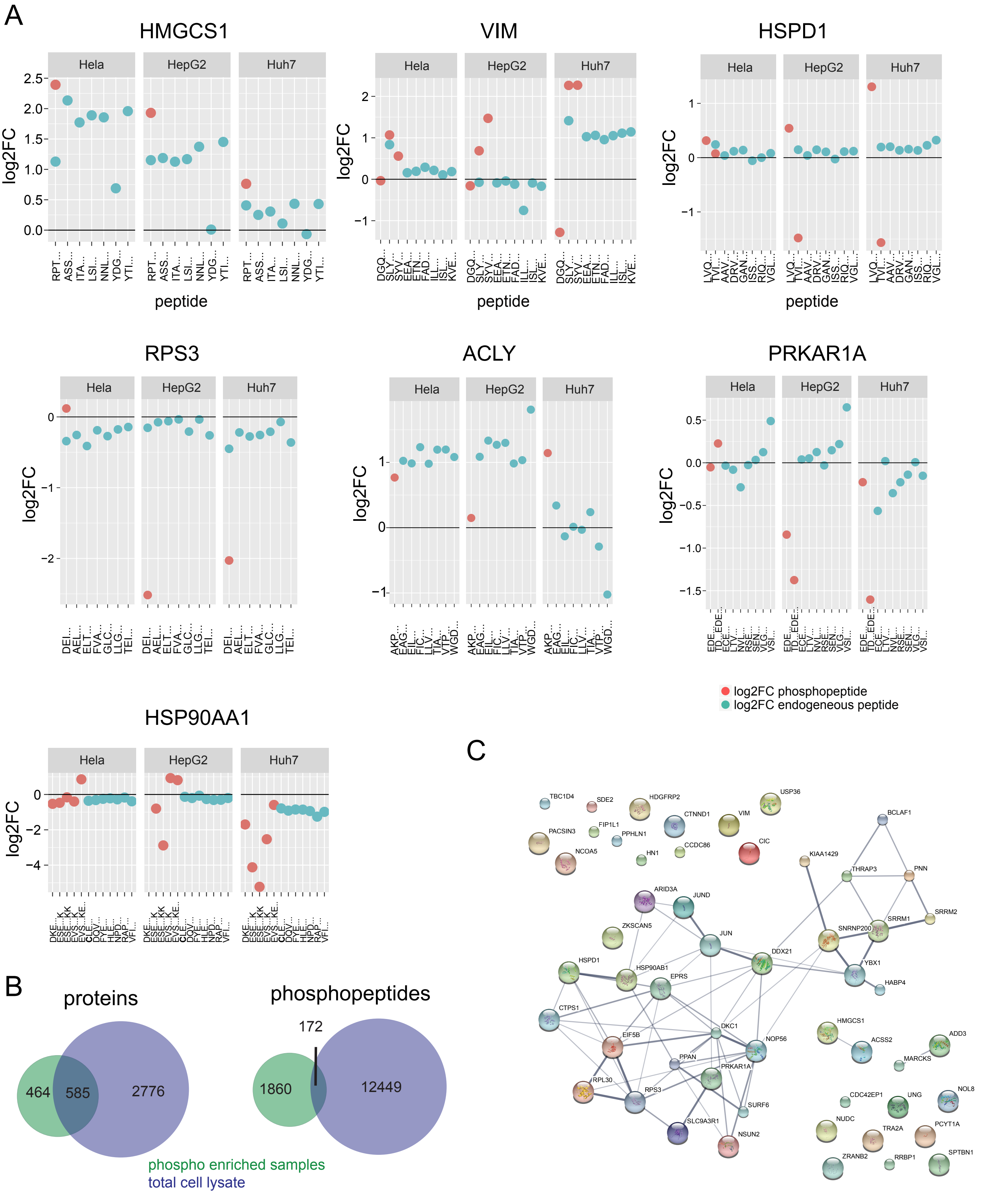

Figure S3: Phosphopeptides whose abundance was affected by sterol depletion, Related to Figure 3D
A) Change in peptide abundance upon treatment with LPDS + atorvastatin compared to untreated cells (red: phosphoenriched peptides LPDS + 1μM atorvastatin versus untreated, blue: total cell lysate LPDS + 1μM and 5μM atorvastatin compared to untreated and DMSO treated cells). B) Overlap between proteins and peptides measured in total cell lysate and phospho- enriched samples. C) Network of phosphoproteins containing phosphosites with differential expression in at least two cell lines connected using the string database (www.string-db.org). Edges represent confidence in interaction based on text mining, experiments, databases, co-expression, neighborhood and gene fusion with minimum confidence of 0.4. See also Table S3.

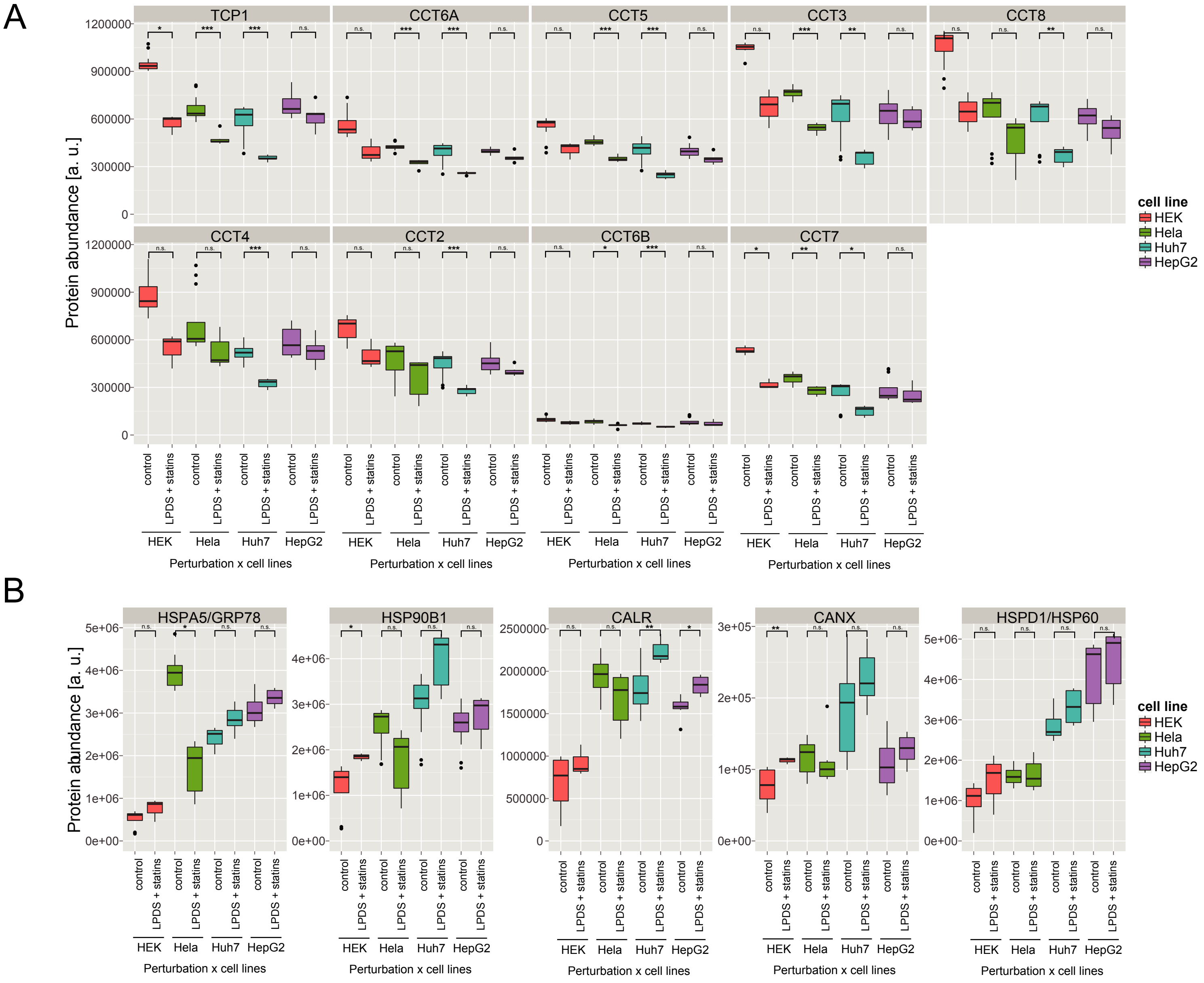

Figure S4: Change of abundance of chaperones and chaperonins upon sterol depletion, Related to Figure 4
A) Abundance of different subunits of the chaperonin TriC/CCT in control treated cells (untreated, DMSO or EtOH) and LPDS + statin treated cells (1μM atorvastatin and 5μM atorvastatin, except for HEK only 1μM atorvastatin). B.) Shown are the data for other cellular chaperones. A + B) P-values for differential expression was determined using an unpaired t-test (n=3-12; n.s. if p-value > 0.01, * p-value < 0.01, ** p-value < 0.001, *** p-value < 1E-4). See also Table S1.

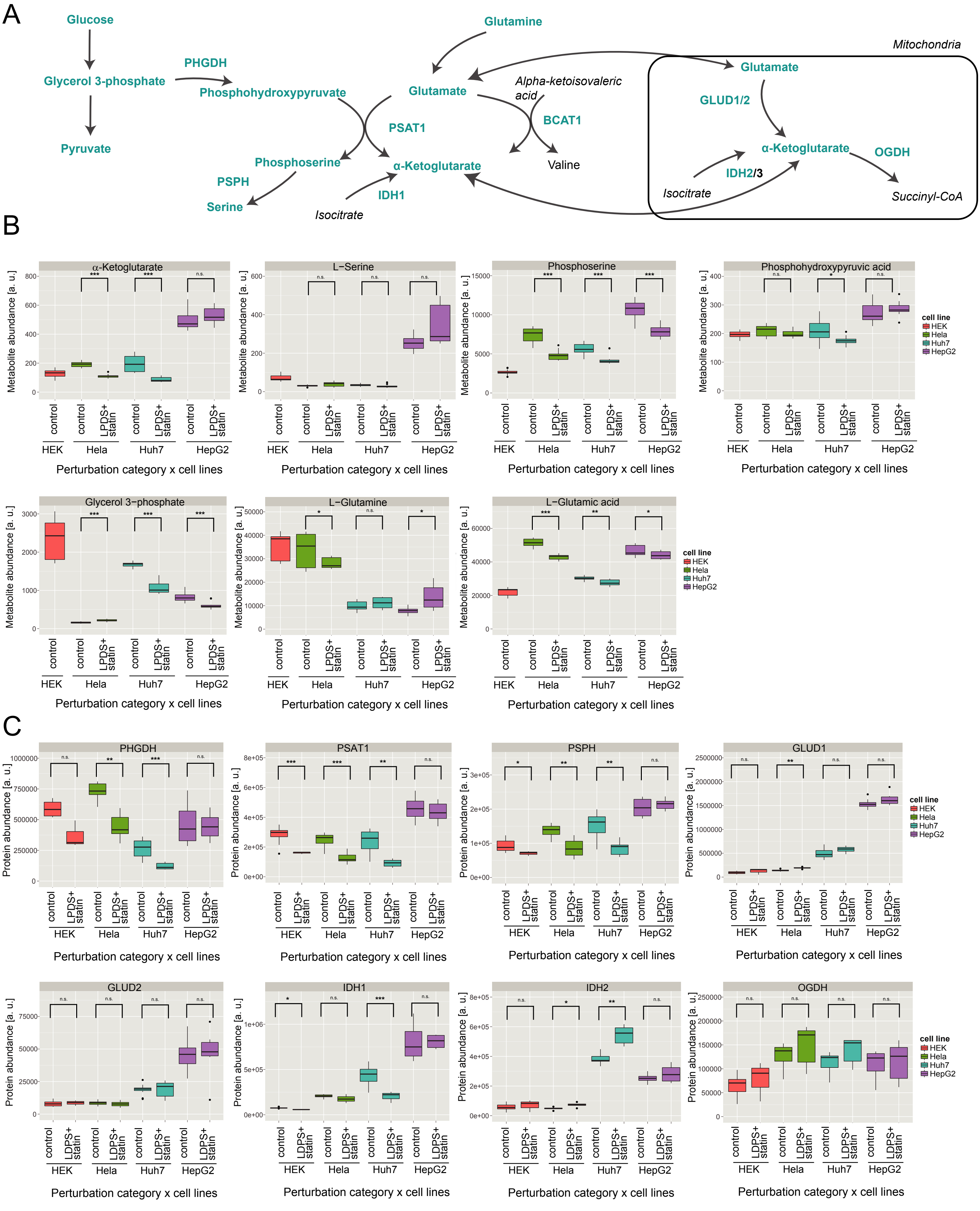

Figure S5: Protein and metabolites from the serine biosynthesis pathway, Related to Figure 4

A) Reaction scheme of the serine biosynthesis pathway with proteins and metabolites quantified labeled in color and bold. Shown is the abundance for selected metabolites (B) and proteins (C) for the control and LPDS + atorvastatin treated conditions (control: untreated, DMSO, EtOH treated cells; LPDS + statin: LPDS + 1µM statin, LPDS + 5µM statin treated cells.) P-value for differential signal was determined using an unpaired t-test: n.s. p-value > 0.01, * p-value < 0.01, ** p-value < 0.001, *** p-value < 1E-4).

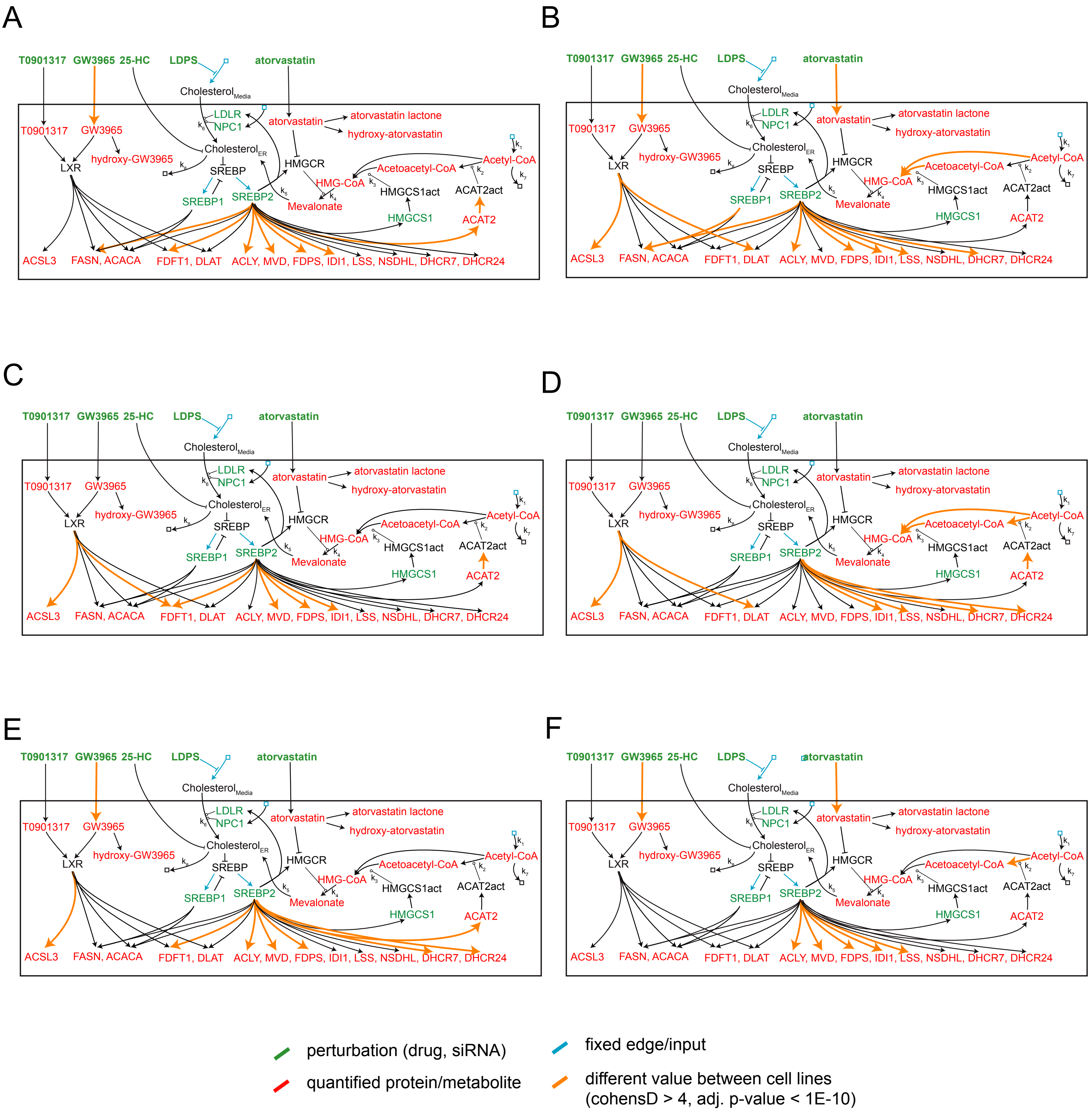

Figure S6: Differences in cell line-specific models, Related to Figure 6A

Prior knowledge network used for modeling. Arrows indicate positive interaction, inverted T indicate inhibitory interaction between nodes (proteins, metabolites or activities). Metabolic reaction are labeled with a rate constant k1-7. Blue edges and inputs have been fixed (see also Methods). Edges that show a significant change between cell lines are depicted in orange. A) Huh7 versus HepG2, B) Huh7 versus Hela, C) HepG2 versus Hela, D) Huh7 versus HEK293, E) HepG2 versus HEK293, F) HEK 293 versus Hela. See also Table S7.
