# Supplementary material for: Systems Pharmacology Dissection of Cholesterol Regulation Reveals Determinants of Large Pharmacodynamic Variability between Cell Lines
Source: Cell Syst. 2017 Dec 27;5(6):604–619.e7. doi: 10.1016/j.cels.2017.11.002 (PMC5747350; doi:10.1016/j.cels.2017.11.002)
Supplement: Data S1. Modeling Scripts, Related to Figure 6 — Zipped file containing the scripts and code used for modeling and to produce the figures related to modeling. [file mmc8.zip › CNOR_CholReg/CNORode_2.13.18.tar.gz!/CNORode/inst/doc/CNORode-vignette.pdf]

Training Signalling Pathway Maps to Biochemical Data with Logic-Based Ordinary Differential Equations

David Henriques[1,2] and Thomas Cokelaer *[1]

[1]European Bioinformatics Institute, Saez-Rodriguez group, Cambridge, United Kingdom
[2]Instituto de Investigaciones Marinas-CSIC, Vigo, Spain

October 13, 2015

Contents

1 Introduction **1**

2 Installation **2**

3 Quick Start **2**

1 Introduction

Mathematical models are used to understand protein signalling networks so as to provide an integrative view of pharmacological and toxicological processes at molecular level. *CellNOptR* [1] is an existing package (see `http://bioconductor.org/packages/release/bioc/html/CellNOptR.html`) that provides functionalities to combine prior knowledge network (about protein signalling networks) and perturbation data to infer functional characteristics (of the signalling network). While *CellNOptR* has demonstrated its ability to infer new functional characteristics, it is based on a boolean formalism where protein species are characterised as being fully active or inactive. In contrast, logic-based ordinary differential equations allow a quantitive description of a given Boolean model.

The method used here was first published by Wittmann et al. [9] by the name of *odefy*. For a detailed description of the methodology the user is adressed to [9] and for a published application example to [6].

This package implements the Odefy method and focus mainly extending the *CellNOptR* capabilities in order to simulate and calibrate logic-based ordinary differential equation model. We provide direct and easy to use interface to optimization methods available in R such as *eSSR* [7] (enhanced Scatter Search Metaheuristic for R) and an R genetic algorithm implementation by the name of *genalg* in order to perform parameter estimation. Additionally we were specially careful in tackling the main computanional bottlenecks by implementing CNORode simulation engine in the C language using the *CVODES* library [10].

This brief tutorial shows how to use *CNORode* using as a starting point a Boolean model and a dataset consisting in a time-series of several proteins.

*cokelaer@ebi.ac.uk

2 Installation

CNORode depends on *CellNOptR* and *genalg*, which are 2 bioconductor packages. Therefore, in order to install *CNORode*, open a R session and type:

```
source("http://bioconductor.org/biocLite.R")
biocLite("CNORode")
```

It may take a few minutes to install all dependencies if you start from scratch (i.e, none of the R packages are installed on your system). Note also that under Linux system, some of these packages necessitate the R-devel package to be installed (e.g., under Fedora type *sudo yum install R-devel*).

Additionally, for parameter estimation we recommend the use of *eSSR*. This algorithm algorithm is part of the MEIGOR toolbox. Although this package is not available on BioConductor, it can be downloaded from http://www.ebi.ac.uk/saezrodriguez/cno/downloads.html. Once downloaded, install MEIGOR by typing:

```
install.packages("MEIGOR_0.99.3_svn2444.tar.gz",type="source")
```

Note "~/" should point to the directory where the file is saved.

Finally, once *CNORode* is installed you can load it by typing:

```
library(CNORode)
```

3 Quick Start

In this section, we provide a quick example on how to use *CNORode* to find the set of continuous parameters which minimize the squared difference between a model simulation and the experimental data.

Since here we will not be modifying the model structure as opposed to *CellNOptR* we will use a model that already contains AND type gates. Such model can be for instance the result of calibrating a *prior knowledge network*(PKN) with *CellNOptR*. Please note that a PKN can also be used as Boolean model which will contain only OR type gates.

Detailed information about the model used here (ToyModelMMB_FeedbackAnd) and additional models can be found at:

http://www.ebi.ac.uk/~cokelaer/cno/doc/sampleModels/

The example used here is shipped with the CNORode. In order to load the data and model you should type the following commands:

```
        library(CNORode)
        model=readSIF(system.file("doc", "ToyModelMMB_FeedbackAnd.sif",
                package="CNORode"));
        cno_data=readMIDAS(system.file("doc", "ToyModelMMB_FeedbackAnd.csv",
                package="CNORode"));
        cnolist=makeCNOlist(cno_data,subfield=FALSE);
```

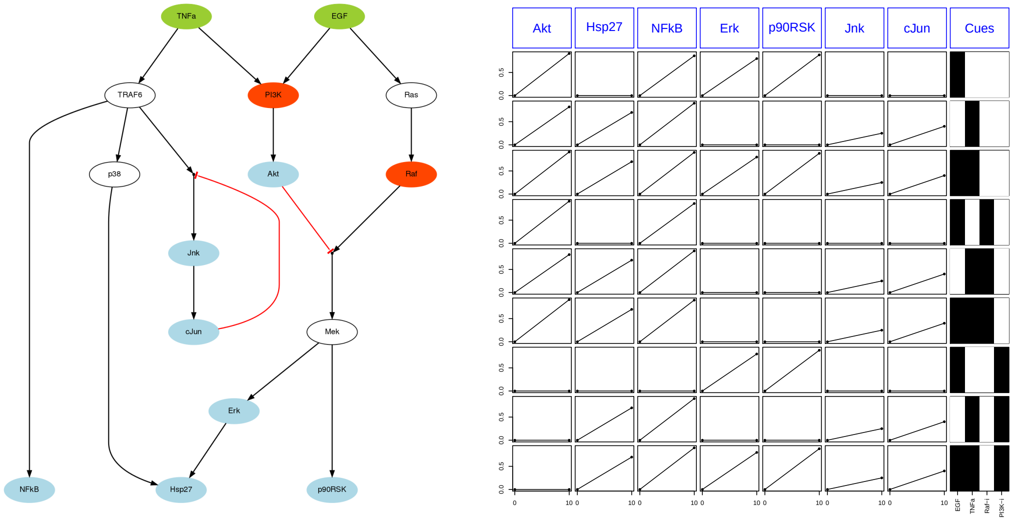

Figure 1: The used model(left panel). A plot from the data, resulting from the *plotCNOlist* function (right panel).

The structure from the CNOlist and the Model object is exactly the same as used in the *CellNOptR* and therefore for a detailed explanation about these structure we direct the reader to the *CellNOptR* manual.

In order to simulate the model and perform parameter estimation we first need to create a list with the ODE parameters associated with each dynamic state as described in [9]. Each dynamic state will have a τ parameter, as many n and k parameters as inputs. Although the default is to use the normalized Hill function it also possible to use the standard Hill or even not to use any transfer function at all. To illustrate the shape of the equations associated to each dynamic state and the meaning of each parameter, let us show the differential of *Mek*:

$$\dot{Mek} = \left[\left(1 - \frac{Akt^{n_1}/({k_1}^{n_1} + Akt^{n_1})}{1/({k_1}^{n_1}+1)}\right) \cdot \left(\frac{Raf^{n_2}/({k_2}^{n_2} + Raf^{n_2})}{1/({k_2}^{n_2}+1)}\right) - Mek\right] \cdot \tau_{Mek}$$

To create a list of ODE parameters we will typically use the *createLBodeContPars* function:

```
ode_parameters=createLBodeContPars(model, LB_n = 1, LB_k = 0.1,
         LB_tau = 0.01, UB_n = 5, UB_k = 0.9, UB_tau = 10, default_n = 3,
         default_k = 0.5, default_tau = 1, opt_n = TRUE, opt_k = TRUE,
         opt_tau = TRUE, random = FALSE)
```

This function creates a general structure where the ODE parameters are ordered according to the model. Some tweaks have been added in order to ease tasks we have found to be common, nevertheless you can edit several attributes manually. If you print the *ode_parameters* list you will see the following attributes.

```
print(ode_parameters)
```

```
$parNames
 [1] "cJun_n_Jnk"   "cJun_k_Jnk"   "TRAF6_n_Jnk"  "TRAF6_k_Jnk"
 [5] "tau_Jnk"      "Mek_n_Erk"    "Mek_k_Erk"    "tau_Erk"
 [9] "Jnk_n_cJun"   "Jnk_k_cJun"   "tau_cJun"     "TRAF6_n_p38"
[13] "TRAF6_k_p38"  "tau_p38"      "TNFa_n_TRAF6" "TNFa_k_TRAF6"
[17] "tau_TRAF6"    "PI3K_n_Akt"   "PI3K_k_Akt"   "tau_Akt"
[21] "Ras_n_Raf"    "Ras_k_Raf"    "tau_Raf"      "TNFa_n_PI3K"
[25] "TNFa_k_PI3K"  "EGF_n_PI3K"   "EGF_k_PI3K"   "tau_PI3K"
[29] "EGF_n_Ras"    "EGF_k_Ras"    "tau_Ras"      "Akt_n_Mek"
[33] "Akt_k_Mek"    "Raf_n_Mek"    "Raf_k_Mek"    "tau_Mek"
[37] "Erk_n_Hsp27"  "Erk_k_Hsp27"  "p38_n_Hsp27"  "p38_k_Hsp27"
[41] "tau_Hsp27"    "TRAF6_n_NFkB" "TRAF6_k_NFkB" "tau_NFkB"
[45] "Mek_n_p90RSK" "Mek_k_p90RSK" "tau_p90RSK"

$parValues
 [1] 3.0 0.5 3.0 0.5 1.0 3.0 0.5 1.0 3.0 0.5 1.0 3.0 0.5 1.0 3.0 0.5
[17] 1.0 3.0 0.5 1.0 3.0 0.5 1.0 3.0 0.5 3.0 0.5 1.0 3.0 0.5 1.0 3.0
[33] 0.5 3.0 0.5 1.0 3.0 0.5 3.0 0.5 1.0 3.0 0.5 1.0 3.0 0.5 1.0

$index_opt_pars
 [1]  1  2  3  4  5  6  7  8  9 10 11 12 13 14 15 16 17 18 19 20 21 22
[23] 23 24 25 26 27 28 29 30 31 32 33 34 35 36 37 38 39 40 41 42 43 44
[45] 45 46 47

$index_n
 [1]  1  3  6  9 12 15 18 21 24 26 29 32 34 37 39 42 45

$index_k
 [1]  2  4  7 10 13 16 19 22 25 27 30 33 35 38 40 43 46

$index_tau
 [1]  5  8 11 14 17 20 23 28 31 36 41 44 47

$LB
 [1] 1.00 0.10 1.00 0.10 0.01 1.00 0.10 0.01 1.00 0.10 0.01 1.00 0.10
[14] 0.01 1.00 0.10 0.01 1.00 0.10 0.01 1.00 0.10 0.01 1.00 0.10 1.00
[27] 0.10 0.01 1.00 0.10 0.01 1.00 0.10 1.00 0.10 0.01 1.00 0.10 1.00
[40] 0.10 0.01 1.00 0.10 0.01 1.00 0.10 0.01

$UB
 [1]  5.0  0.9  5.0  0.9 10.0  5.0  0.9 10.0  5.0  0.9 10.0  5.0  0.9
[14] 10.0  5.0  0.9 10.0  5.0  0.9 10.0  5.0  0.9 10.0  5.0  0.9  5.0
[27]  0.9 10.0  5.0  0.9 10.0  5.0  0.9  5.0  0.9 10.0  5.0  0.9  5.0
[40]  0.9 10.0  5.0  0.9 10.0  5.0  0.9 10.0
```

Typically before running an optimization run you will want to choose which type of parameters you want to optimize. The field *index_opt_pars* defines which parameters are meant to be optimized. In the *createLBodeContPars*, if you choose *opt_tau* as $TRUE$ all τ parameters will be added to the index_opt_pars array, the same idea is valid for n and k parameters.

It is also possible to choose default values for lower and upper bounds for the parameters of a given type, e.g. τ (*LB_tau* and *UB_tau*), as well as a default initial value for such parameters.

Once we have the ODE parameters structure we are ready to run a simulation or optimization process. To run a simulation we can use the *getLBodeModel* or *getLBodeDataSim*, depending on if we want to simulate only the signals present in the CNOlist object or all the species in the model. Additionally *plotLBodeDataSim* or *plotLBodeModelSim* will also return the values of a model simulation while plotting the same values. In figure 2, we use plotLBodeModelSim to plot all the experiments sampled in 5 different instants (*timeSignals*).

```
modelSim=plotLBodeModelSim(cnolist, model, ode_parameters,
          timeSignals=seq(0,2,0.5));
```

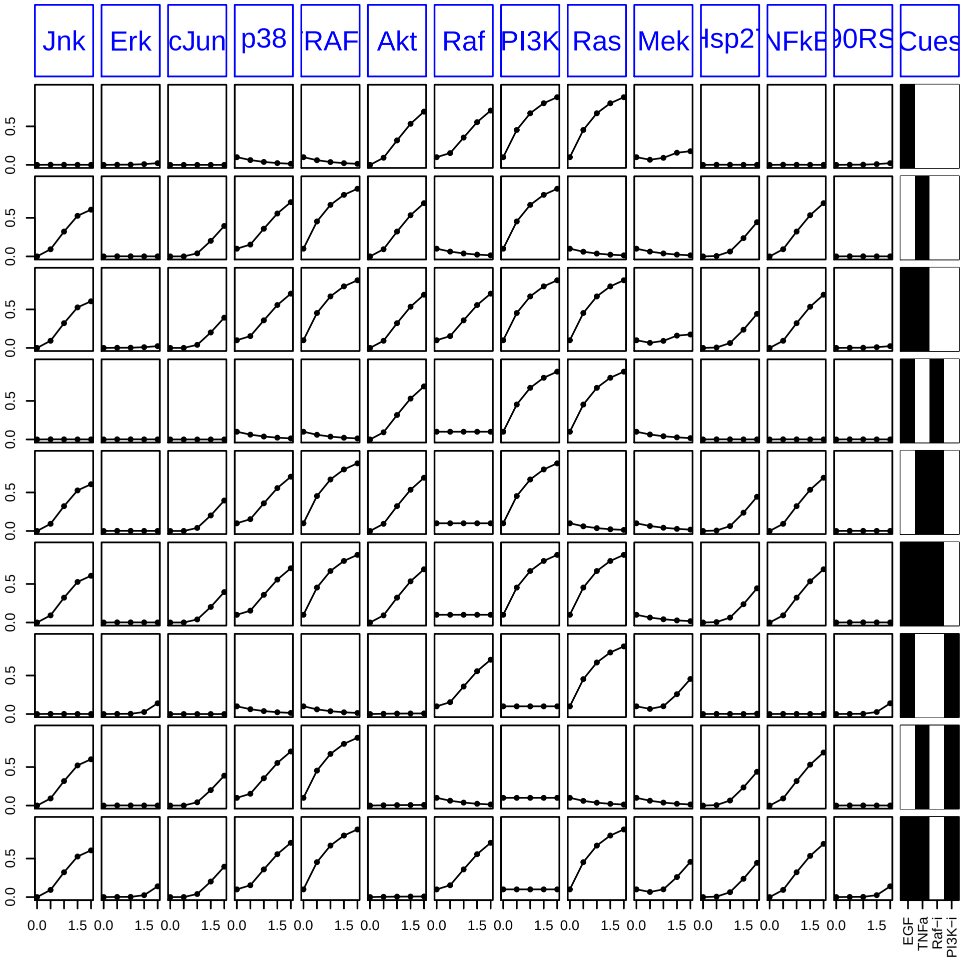

Figure 2: A model simulation plotted with *plotLBodeModelSim* ..

As previously mentioned, we provide two optimization algorithms that allow parameter estimation Both of these algorithms have specific parameters that can be tunned on each specific problem (please check CNORode manual for detailed information). For instance, in order to run the genetic algorithm for 10 iterations and a population of size of 10, we can use the following code:

```
initial_pars=createLBodeContPars(model, LB_n = 1, LB_k = 0.1,
         LB_tau = 0.01, UB_n = 5, UB_k = 0.9, UB_tau = 10, random = TRUE)
#Visualize initial solution
simulatedData=plotLBodeFitness(cnolist, model,initial_pars)
paramsGA = defaultParametersGA()
paramsGA$maxStepSize = 1
paramsGA$popSize = 50
paramsGA$iter = 100
paramsGA$transfer_function = 2
opt_pars=parEstimationLBode(cnolist,model,ode_parameters=initial_pars,
         paramsGA=paramsGA)
#Visualize fitted solution
simulatedData=plotLBodeFitness(cnolist, model,ode_parameters=opt_pars)
```

```
simulatedData=plotLBodeFitness(cnolist, model,initial_pars)
```

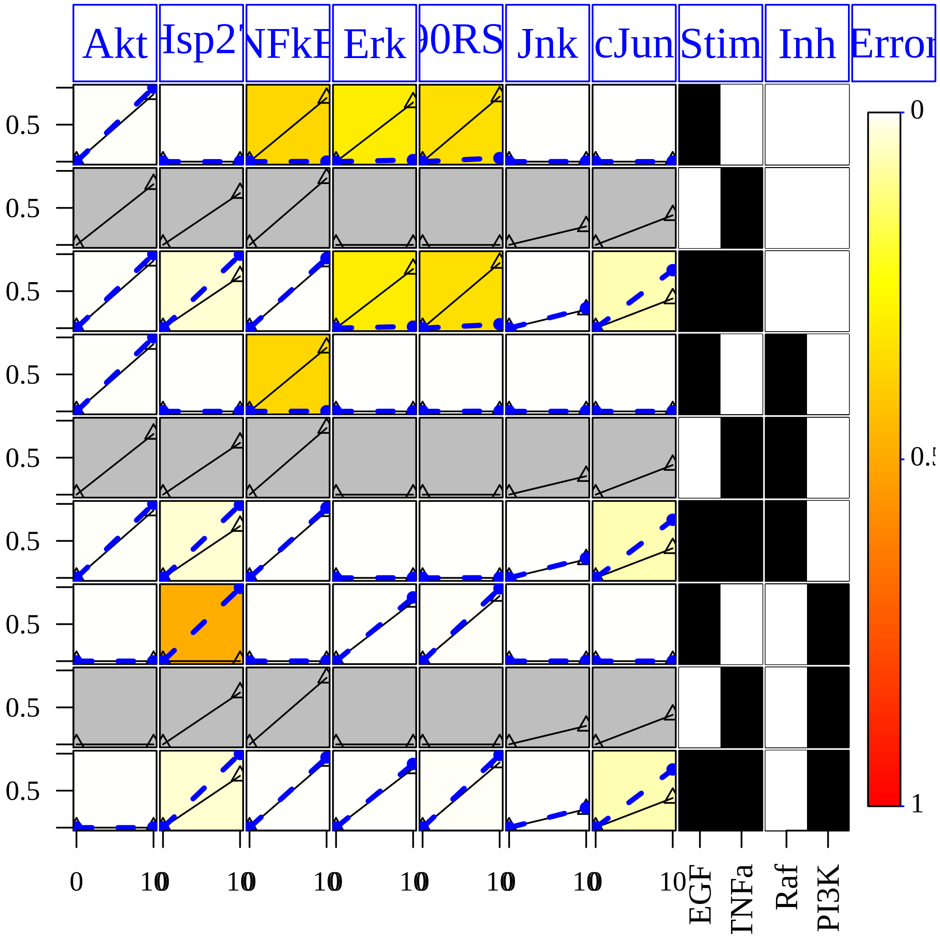

Figure 3: The initial solution before optimization. Each row corresponds to an experiment with a particular combination of stimuli and inhibitors. The columns correspond to the measured values (triangles) and the simulated values (dashed blue lines) from a given signal. The background color gives an indication of squared difference where red means high error and white low error.

```
simulatedData=plotLBodeFitness(cnolist, model,ode_parameters=opt_pars)
```

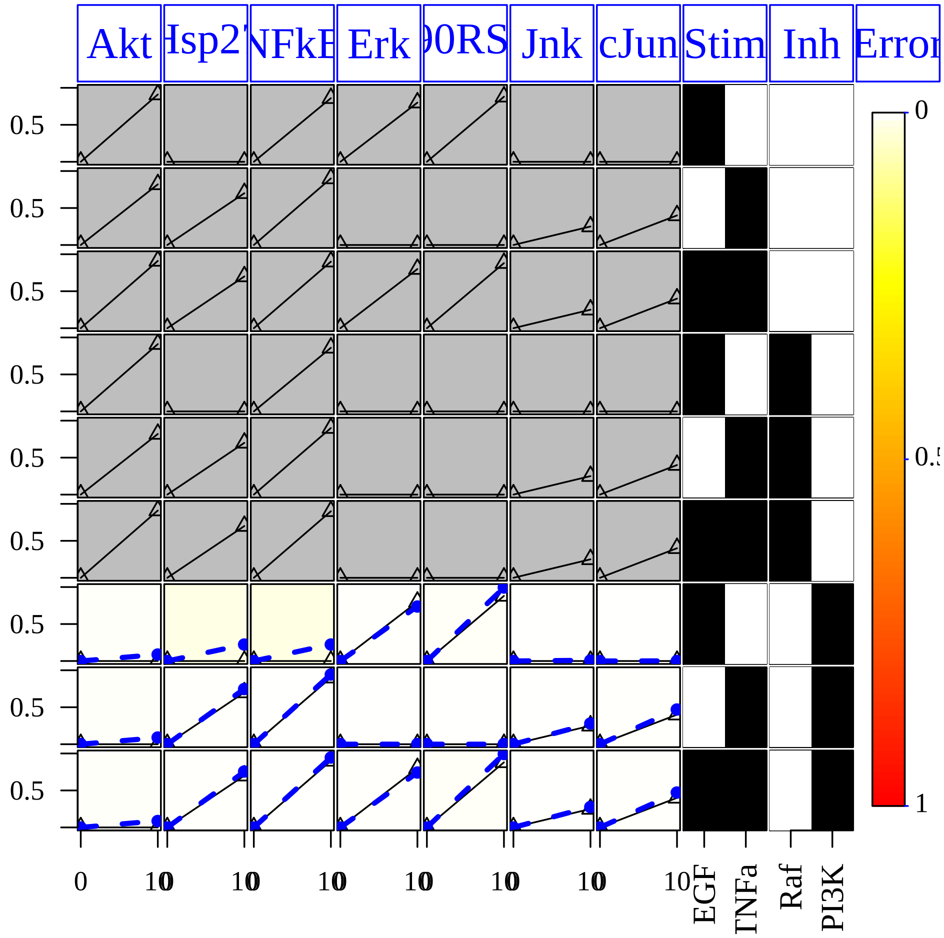

Figure 4: A solution obtained by optimization with a genetic algorithm.Each row corresponds to an experiment with a particular combination of stimuli and inhibitors. The columns correspond to the measured values (triangles) and the simulated values (dashed blue lines) from a given signal. The background color gives an indication of squared difference where red means high error and white low error.

In addition to eSSR and genalg its is fairly easy to use any other continuous optimization algorithm. In the following example we show how to generate and use an the objective function in order to use it with a variant of eSSR(part of MEIGOR package) that uses multiple cpus:

```
library(MEIGOR)
f_hepato<-getLBodeContObjFunction(cnolist, model, initial_pars, indices=NULL,
  time = 1, verbose = 0, transfer_function = 2, reltol = 1e-05, atol = 1e-03,
 maxStepSize = Inf, maxNumSteps = 1e4, maxErrTestsFails = 50, nan_fac = 1)
n_pars=length(initial_pars$LB);
problem<-list(f=f_hepato, x_L=initial_pars$LB[initial_pars$index_opt_pars],
         x_U=initial_pars$UB[initial_pars$index_opt_pars]);
#Source a function containing the options used in the CeSSR publication
 source(system.file("benchmarks","get_paper_settings.R",package="MEIGOR"))
#Set max time as 20 seconds per iteration
opts<-get_paper_settings(20);
Results<-CeSSR(problem,opts,Inf,Inf,3,TRUE,global_save_list=c('cnolist','model',
 'initial_pars'))
```

References

[1] C. Terfve. CellNOptR: R version of CellNOpt, boolean features only. R package version 1.2.0, (2012) http://www.bioconductor.org/packages/release/bioc/html/CellNOptR.html

[2] L.G. Alexopoulos, J. Saez-Rodriguez, B.D. Cosgrove, D.A. Lauffenburger, P.K Sorger.: Networks inferred from biochemical data reveal profound differences in toll-like receptor and inflammatory signaling between normal and transformed hepatocytes. Molecular & Cellular Proteomics: MCP **9**(9), 1849–1865 (2010).

[3] M.K. Morris, I. Melas, J. Saez-Rodriguez. Construction of cell type-specific logic models of signalling networks using CellNetOptimizer. *Methods in Molecular Biology: Computational Toxicology*, Ed. B. Reisfeld and A. Mayeno, Humana Press.

[4] M.K. Morris, J. Saez-Rodriguez, D.C. Clarke, P.K. Sorger, D.A. Lauffenburger. Training Signaling Pathway Maps to Biochemical Data with Constrain ed Fuzzy Logic: Quantitative Analysis of Liver Cell Responses to Inflammatory Stimuli. *PLoS Comput Biol.* 7(3) (2011) : e1001099.

[5] J. Saez-Rodriguez, L. Alexopoulos, J. Epperlein, R. Samaga, D. Lauffenburger, S. Klamt and P.K. Sorger. Discrete logic modelling as a means to link protein signalling networks with functional analysis of mammalian signal transduction. *Molecular Systems Biology*, 5:331, 2009.

[6] Dominik Wittmann, Jan Krumsiek, Julio S. Rodriguez, Douglas Lauffenburger, Steffen Klamt, and Fabian Theis. Transforming boolean models to continuous models: methodology and application to t-cell receptor signaling. BMC Systems Biology, 3(1):98+, September 2009.

[7] Egea, J.A., Maria, R., Banga, J.R. (2010) An evolutionary method for complex-process optimization. Computers & Operations Research 37(2):315324.

[8] Egea, J.A., Balsa-Canto, E., Garcia, M.S.G., Banga, J.R. (2009) Dynamic optimization of nonlinear processes with an enhanced scatter search method. Industrial & Engineering Chemistry Research 49(9): 43884401.

[9] Jan Krumsiek, Sebastian Polsterl, Dominik Wittmann, and Fabian Theis. Odefy - from discrete to continuous models. BMC Bioinformatics, 11(1):233+, 2010.

[10] R. Serban and A. C. Hindmarsh ”CVODES: the SensitivityEnabled ODE Solver in SUNDIALS,” Proceedings of IDETC/CIE 2005, Sept. 2005, Long Beach, CA. Also available as LLNL technical report UCRLJP200039.
