# Supplementary material for: Systems Pharmacology Dissection of Cholesterol Regulation Reveals Determinants of Large Pharmacodynamic Variability between Cell Lines
Source: Cell Syst. 2017 Dec 27;5(6):604–619.e7. doi: 10.1016/j.cels.2017.11.002 (PMC5747350; doi:10.1016/j.cels.2017.11.002)
Supplement: Data S1. Modeling Scripts, Related to Figure 6 — Zipped file containing the scripts and code used for modeling and to produce the figures related to modeling. [file mmc8.zip › CNOR_CholReg/CNORode_2.13.18.tar.gz!/CNORode/vignettes/data_ToyModelMMB_FeddbackAnd.pdf]

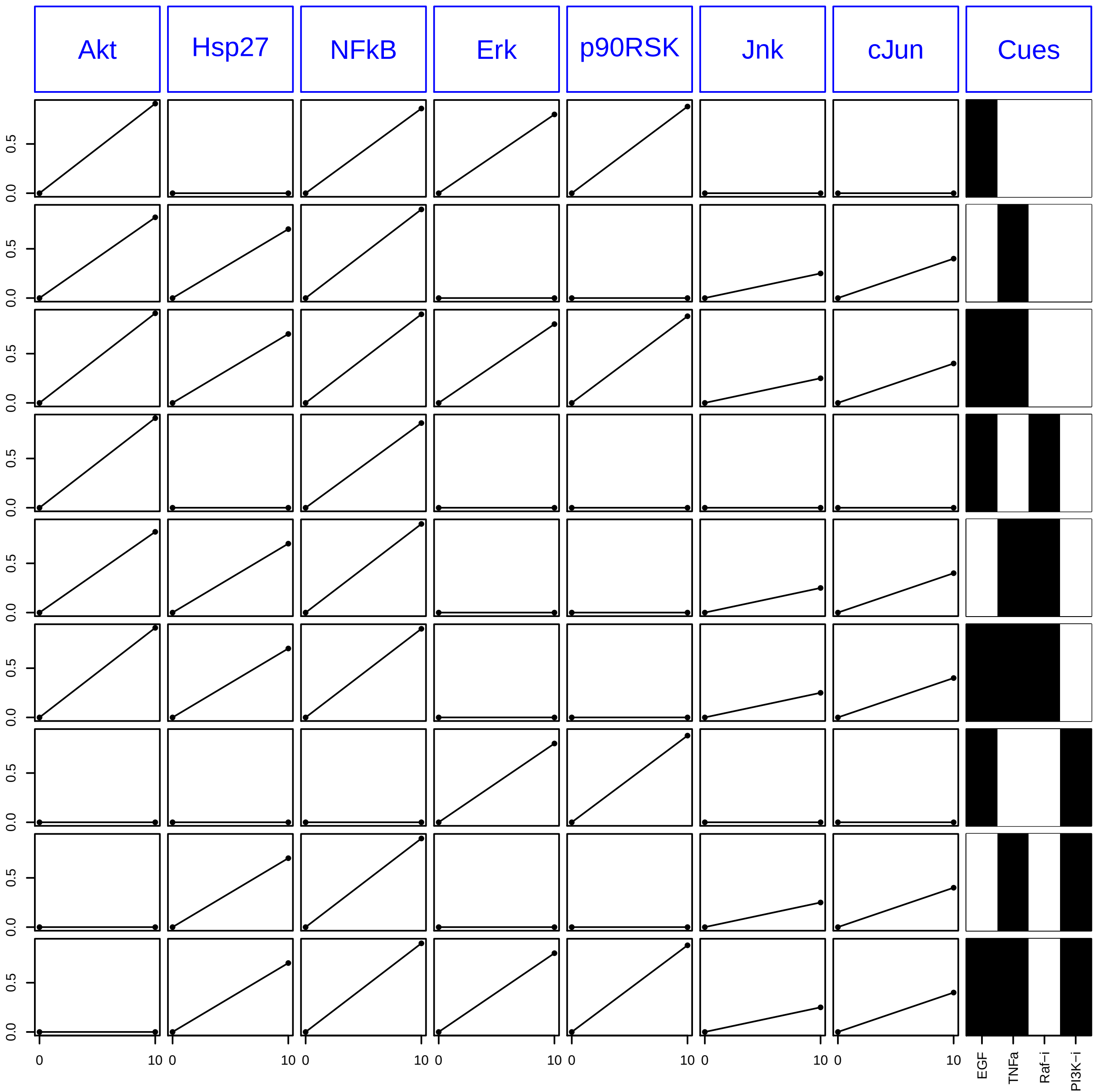
Akt
Hsp27
NFkB
Erk
p90RSK
Jnk
cJun
Cues
0.0
0.5
0
10
EGF
TNFa
Raf−i
PI3K−i
