# Supplementary material for: Systems Pharmacology Dissection of Cholesterol Regulation Reveals Determinants of Large Pharmacodynamic Variability between Cell Lines
Source: Cell Syst. 2017 Dec 27;5(6):604–619.e7. doi: 10.1016/j.cels.2017.11.002 (PMC5747350; doi:10.1016/j.cels.2017.11.002)
Supplement: Data S1. Modeling Scripts, Related to Figure 6 — Zipped file containing the scripts and code used for modeling and to produce the figures related to modeling. [file mmc8.zip › CNOR_CholReg/README.docx]

# Documentation of codes used for modeling cholesterol regulation in the manuscript “Systems pharmacology dissection of cell specific cholesterol regulation mechanisms”

Created by Peter Blattmann and David Henriques

Last revised: February 17, 2017

Contacts: blattmann@imsb.biol.ethz.ch and davidh@iim.csic.es

This code serves the purpose of reproducing the modeling results from the paper “*Systems pharmacology dissection of cell specific cholesterol regulation mechanisms”*. A modified version of the CNORode package is distributed along with the scripts needed to reproduce all results and figures. The model is encoded in the rhsODE.c and a network SIF file. The code is not distributed with the purpose of being a user-friendly software. It requires a LINUX cluster as well as some knowledge in LINUX and R programming.

### REQUIREMENTS AND INSTALLATION NOTES

**LINUX:** The code provided here is designed to run in a LINUX cluster using the LSF queue system. Running the whole pipeline in a different OS may require major efforts. Some tasks are automated with the help of shell scripts and a queue system. Cluster configurations vary a lot. However with moderate effort, the code provided here could be adapted to run in other LINUX machines.

### INSTALLING DEPENDENCIES

**Install package truncnorm**

install.packages('truncnorm')

**Install MEIGOR manually**

install.packages('MEIGOR',repos=NULL,type='src')

Note: Along with the rest of the code provided here we ship a version of the optimization package MEIGOR. This modified version has a maximum limit of 40000 function evaluations for the local solver DHC. File MEIGOR/R/ssm_localsolver.R

**Install graph**

source("http://bioconductor.org/biocLite.R")

biocLite("graph")

**Install RBGL**

source("http://bioconductor.org/biocLite.R")

biocLite("RBGL")

Update all/some/none? [a/s/n]: n

**Install Rgraphviz**

biocLite("Rgraphviz")

**Install CellNOptR**

source("http://bioconductor.org/biocLite.R")

biocLite("CellNOptR")

Update all/some/none? [a/s/n]: n

## CODE AND RESULTS ORGANIZATION

The code and results are organized into 4 folders: 1_input_data, 2_cluster_files, 3_results and 4_analysis.

**1_Input_data**

The experimental results in the MIDAS forma and prior knowledge network in SIF format.

**2_cluster_files**

The files used to run the parameter optimization/bootstrap procedure. The optimization is run with the different subsets of data obtained by bootstrapping from the replicates of the experimental data. The bootstrap is applied to 4 different cell lines (Hek, Hela, HepG2, Huh7).

2_cluster_files\**launch_opt_bootstrap.sh:** Master shell script to launch the bootstrap for all cell-lines. This script is written to work with the LSF cluster system. Modifications to your particular environment might be necessary. The number of data realizations for the bootstrap is encoded in this script and is by default set to 100. Additionally, there is a an associated shell script for each cell-line:

2_cluster_files\**run_peter_pert_bootstrap_Hek.sh**

2_cluster_files\**run_peter_pert_bootstrap_Hela.sh**

2_cluster_files\**run_peter_pert_bootstrap_HepG2.sh**

2_cluster_files\**run_peter_pert_bootstrap_Huh7.sh**

Further, there is a an associated R script for each cell-line:

2_cluster_files\Rscripts\**run_clust_opt_pert_bootstrap_Hek.R**

2_cluster_files\Rscripts\**run_clust_opt_pert_bootstrap_Hela.R**

2_cluster_files\Rscripts\**run_clust_opt_pert_bootstrap_HepG2.R**

2_cluster_files\Rscripts\**run_clust_opt_pert_bootstrap_Huh7.R**

Finally, a set of functions, common to all cell-lines is encoded in:

2_cluster_files\Rscripts\**run_opt_pert_functions.R**

**3_results**

The results from the bootstrap saved as RData files. For the sake of reproducibility random seeds are also stored.

**4_analysis**

This folder contains the scripts used to analyze the results in the “3_results” folder generated using a computer cluster and produce the figures seen in the manuscript.

**1_processRes.R**: This processes the results from the Cluster output and writes into a .RData file.

**2_plotting_different_edges.R**: Plot pdf files for the edges.

**3_statisticaltest.R**: Compute the statistical comparison between the different models

**4_Ensemble_plots.R**: Plot the ensemble of trajectories of all models.

**5_predictions.R**: Functions to make the plots in Figure 7D and E

## WORFLOW

1) Install all the dependencies.
2) Install the CNORode package: R CMD INSTALL CNORode_2.13.18.tar.gz
3) Launch **launch_opt_bootstrap.sh.** Don’t forget the diverse shell (.sh) scripts might need permission for execution.
4) Wait that all the bootstrap/optimization instances finish.
5) Run ( source(‘…’) ) the scripts for analysis the order marked: 1_processRes.R, 2_plotting_different_edges.R, 3_statisticaltest.R and 4_Ensemble_plots.R.
